# Intestinal epithelial damage-derived mtDNA activates STING-IL12 axis in dendritic cells to promote colitis

**DOI:** 10.7150/thno.96184

**Published:** 2024-07-16

**Authors:** Yajie Cai, Shuo Li, Yang Yang, Shuni Duan, Guifang Fan, Jinzhao Bai, Qi Zheng, Yiqing Gu, Xiaojiaoyang Li, Runping Liu

**Affiliations:** 1 School of Chinese Materia Medica, Beijing University of Chinese Medicine, 11 Bei San Huan Dong Lu, Beijing, 100029, China.; 2 School of Life Sciences, Beijing University of Chinese Medicine, 11 Bei San Huan Dong Lu, Beijing, 100029, China.

**Keywords:** myeloid STING, dendritic cells, IL-12, mtDNA

## Abstract

**Rationale:** The treatment of ulcerative colitis (UC) presents an ongoing clinical challenge. Emerging research has implicated that the cGAS-STING pathway promotes the progression of UC, but conflicting results have hindered the development of STING as a therapeutic target. In the current study, we aim to comprehensively elucidate the origins, downstream signaling and pathogenic roles of myeloid STING in colitis and colitis-associated carcinoma (CAC).

**Methods:** *Tmem173*^fl/fl^*Lyz2-Cre*^ert2^ mice were constructed for inducible myeloid-specific deletion of STING. RNA-sequencing, flow cytometry, and multiplex immunohistochemistry were employed to investigate immune responses in DSS-induced colitis or AOM/DSS-induced carcinogenesis. Colonic organoids, primary bone marrow derived macrophages and dendritic cells, and splenic T cells were used for *in vitro* studies.

**Results:** We observed that myeloid STING knockout in adult mice inhibited macrophage maturation, reduced DC cell activation, and suppressed pro-inflammatory Th1 and Th17 cells, thereby protecting against both acute and chronic colitis and CAC. However, myeloid STING deletion in neonatal or tumor-present mice exhibited impaired immune tolerance and anti-tumor immunity. Furthermore, we found that TFAM-associated mtDNA released from damaged colonic organoids, rather than bacterial products, activates STING in dendritic cells in an extracellular vesicle-independent yet endocytosis-dependent manner. Both IRF3 and NF-κB are required for STING-mediated expression of IL-12 family cytokines, promoting Th1 and Th17 differentiation and contributing to excessive inflammation in colitis.

**Conclusions:** Detection of the TFAM-mtDNA complex from damaged intestinal epithelium by myeloid STING exacerbates colitis through IL-12 cytokines, providing new evidence to support the development of STING as a therapeutic target for UC and CAC.

## Introduction

Ulcerative colitis (UC) is a chronic inflammatory disease characterized by continuous and relapsing mucosal inflammation. Its incidence and prevalence have been rising around the world. Long-standing extensive UC is associated with increased risk of colorectal cancer (CRC), thus contributing to the mortality related to this disorder. However, the etiology and pathogenesis of UC remain poorly understood, and the unsatisfactory responses to current therapies pose significant challenges 1. The abnormal activation of PRRs is closely related to multiple autoimmune diseases, including UC and colitis-associated cancer (CAC) 2. Recently, stimulator of interferon genes (STING) pathway has emerged to be crucial mechanism in the development of UC by sensing foreign or self-origin dsDNA and promoting innate immune defense programs 3, 4. The single-cell RNA-Sequencing data have shown that the expression of cGAS, STING, TBK1, IRF3, type I IFNs and interferon-stimulated genes (ISGs) were upregulated in active UC patients and in multiple colitis murine models 5, 6. It was also reported that the deficiency of STING significantly alleviated intestinal inflammation in mice, while the inflammation was worsened after administration of STING agonist DMXAA 7, 8. Besides, spontaneous chronic colitis and fibrosis occurred in mice with structural activation of STING (known as N153S mice). Myeloid-derived STING has further been recognized to play a vital role in mediating the development of colitis 6. Specific knockout of STING in Lysm^+^ cells and CD11c^+^ cells were found to ameliorate AOM/DSS-induced CAC 7. These data all suggest that the activation of STING pathway, especially STING stimulation in myeloid cells, may involve in the pathogenesis of UC.

However, Yang *et al.* recently found that abrogation of STING in CD4^+^ T cell induced more severe colonic inflammation through increasing the proportion of pathogenic Th1 cell and suppressing the production of the anti-inflammatory IL-10 9. Another study discovered that congenital knockout STING significantly limited the growth of intestinal barrier and the maturation of local immune homeostasis, leading to immune intolerance and eventual aggravation of colitis 10. Hence, the precise role of STING in colitis remains a subject of debate, and comprehensive and systematic investigations on the function and mechanism of STING are necessary.

The triggers of STING activation and its downstream signaling in UC development is also a mystery. Zhao *et al.* suggested that nuclear DNA and mtDNA packaged by extracellular vesicles (EVs) leaked from damaged intestinal epithelial cells stimulated the STING pathway, thus triggering intestinal inflammation in murine colitis and active human CD 11. However, another report suggested that commensal bacteria and its product c-di-GMP stabilize the expression of STING protein by enhancing K63-linked ubiquitination of STING 6. In terms of STING-mediated signaling, it was demonstrated that myeloid-differentiation primary response protein (MyD88)-dependent IL-1β and IL-18 release exerted pro-inflammatory effects after STING stimulation 7. The accumulation of Th1 effector cells was also proposed as the downstream immune responses upon STING activation, suggesting the involvement of adaptive immunity in the progression of STING-related colitis 6. However, the interaction between myeloid-derived STING pathways and downstream adapt immune responses still lacks evidence.

In the current study, we established DSS-induced acute and chronic colitis models on neonatal and adult *Tmem173*^fl/fl^*Lyz2-Cre*^ert2^ mice, aiming to investigate the exact effects and immune mechanisms of cell-specific and time-dependent STING knockout on UC progression and AOM/DSS-induced CAC. Additionally, we utilized colonic organoids, BMDMs, BMDCs, and primary splenic T cells to elucidate the triggers, signaling transduction, and downstream effectors of STING activation in regulating native and adaptive immune responses during colitis.

## Materials and methods

### Animals

C57BL/6N, *Tmem173*^fl/fl^ mice, and *Lyz2-Cre*^ert2^ mice were purchased from Shanghai Model Organisms Center, Inc. (Shanghai, China). *Tmem173*^fl/fl^ mice were crossed with *Lyz2-Cre*^ert2^ mice to obtain *Tmem173*^fl/fl^*Lyz2-Cre*^ert2^ mice (referred as *Tmem173*^iΔmye^ mice). *Tmem173*^-/-^ mice were kind gifts from Prof. Yao Wang at Beijing University of Chinese Medicine. All genetically modified mice were on the C57BL/6N background. All animal studies were conducted in accordance with the guidelines approved by the Institutional Animal Care and Use Committee at Beijing University of Chinese Medicine guidelines (BUCM-4-2022012001-1096).

### Tamoxifen-induced myeloid-specific deletion of *Tmem173*

To achieve conditional knockout of *Tmem173* in the myeloid cells of mice, 5 to 6-week-old or 6 to 8-week-old *Tmem173*^fl/fl^ mice and* Tmem173*^fl/fl^*Lyz2-Cre*^ert2^ mice were injected intraperitoneally with 120 mg/kg tamoxifen (TAM; ADAMAS, Basel, Switzerland) dissolved in corn oil every other day for 5 times. In the chronic DSS colitis model and AOM/DSS-P groups, we maintained TAM induction twice a week to ensure long-term myeloid-specific STING knockout during the experiments. Neonatal *Tmem173*^fl/fl^ mice and* Tmem173*^iΔmye^ mice were injected with the same dose of TAM into the stomach for 5 continuous days. Genotyping was performed when the mice were four-week-old, following the guidance of Direct Mouse Genotyping Kit (APExBIO, Houston, USA).

### Acute and chronic DSS colitis

C57BL/6N mice, *Tmem173*^-/-^ mice, *Tmem173*^fl/fl^ mice, and* Tmem173*^iΔmye^ mice at the age of 6 to 8-week-old were administered with 2.5%-3% (w/v) dextran sodium sulfate (DSS; MP Biomedicals, California, USA) dissolved in the drinking water for 7 days followed by 1 day water. The control group mice were received regular distilled water. The body weight of mice was monitored every day. Colon tissue was harvested, disease activity index was evaluated, and spleen coefficient (spleen weight divided by the body weight) was calculated at the end of the experiment.

After given one-week acute DSS induction, 6 to 8-week-old* Tmem173*^fl/fl^ mice and* Tmem173*^iΔmye^ mice were administered with a cycle of three days of normal drinking water followed by four days of 2.5%-3% DSS, and this cycle was repeated for three times. The body weight of mice was monitored every day. Colon tissue was harvested, disease activity index was evaluated, and spleen coefficient was calculated at the end of the experiment.

### The extraction of colonic lamina propria cells

The extraction of colonic lamina propria cells was performed under the guidance of lamina propria dissociation kit (Miltenyi, Bergisch Gladbach, Germany). The colonic samples were isolated, flushed, and then cut into 2 to 4 mm fragments. The colonic samples were transferred into 1× HBSS solution containing 5 mM EDTA, 5% fetal bovine serum (FBS) and 1 mM DTT for 20 min at 37 °C under rotation of 12 rpm to remove colonic epithelial cells. This pre-digestion process was repeated twice. Remaining colonic samples was digested by pre-heated 1× HBSS solution containing 5% FBS with mixed enzyme on the gentleMACS Dissociator (Miltenyi, Bergisch Gladbach, Germany). The cell suspension was filtered through 100 μm strainer and then centrifuged at 300×g for 10 min. The cell viability of isolated lamina propria lymphocytes was analyzed by AO/PI dye (Counterstar, Shanghai, China) and was over 80%, which was enough to for further applications.

### The isolation and culture of colonic organoids

The colonic samples were isolated, flushed, and then cut into 2 to 4 mm fragments. Then the colonic fragments were washed by PBS containing 10% FBS (washing solution) till the supernatant was clear. The colonic samples were incubated with 2 mM EDTA for 15 min at 4 °C to digest the colonic crypts, and were then washed by washing solution. This process was repeated twice. The suspension was filtered through a 70 μm strainer and the filtered crypts were collected. The crypts were resuspended with Matrigel and added into 24 well plates at 37 °C for 5-10 min till the Matrigel was solidified, the BM2 medium containing 50 ng/µL EGF, 100 ng/µL Noggin, and 1 µg/µL R-Spondin-1 (culture solution) was added in each well. The fresh culture solution was replaced 2 to 3 times. The colonic organoids needed to be passaged by DMEM/F12 medium containing 1% GlutaMax and 1% HEPES when they became bigger. The organoids were treated with 100 ng/mL TNF-α to induce apoptosis, and the supernatant was collected 2 h later. PI staining was performed to evaluated the injury of colonic organoids.

### The isolation of DNA from the conditioned medium of colonic organoids

DNA isolation from the cultured medium of colonic organoids was performed under the guidance of QIAamp Blood DNA mini kit (QIAGEN, Dusseldorf, Germany). The quality examination and quantification of isolated DNA were performed using Nanodrop (Thermo Scientific, Madison, USA), and then the concentration of DNA in CM was calculated.

### The measurement of mtDNA

Real-time qPCR was employed for the quantification of mtDNA and nuclear DNA using specific primers for mitochondrial genes, including mt-12S1, mt-CO3, mt-CytB, and the nuclear gene rn18S, and results were normalized with rn18S.

### DNA uptake in BMDCs

DNA uptake of BMDCs was measured by DRAQ5 Fluorescent Probe (Thermo, Massachusetts, USA). The supernatant of isolated colonic organoids treated with or without TNF-α was collected and incubated with 5 μM DARQ5 Fluorescent Probe and 10 mM HEPES for 115 min at 37 °C. WT BMDCs were incubated with the DRAQ5-stained conditional medium for 6h and subjected to flow cytometry.

### Immunoprecipitation (IP) of TFAM-associated mtDNA

Protein A/G beads were incubated with IgG and anti-TFAM antibodies overnight at 4 °C with rotator to obtain IgG antibody-coated beads and anti-TFAM antibody-coated beads. Then blank Protein A/G beads with the supernatant of colonic organoids were mixed for 30 min at 4 °C with rotator to block the samples. After centrifugation, the blocked samples were transferred into IgG antibody-coated beads and anti-TFAM antibody-coated beads and then rotated overnight at 4 °C. The obtained precipitate and supernatant were utilized for* in vitro* studies and DNA isolation.

### Quantification and statistical analysis

All values expressed as mean ± SEM were statistically analyzed using GraphPad Prism (version 8.0). Differences among group means were determined with one-way ANOVA with a Tukey *post hoc* test for multi-group comparisons, or an unpaired two-tailed Student's *t* test between two groups. P value of less than 0.05 was considered statistically significant.

## Results

### Myeloid-specific knockout of STING in adult mice ameliorates DSS-induced acute and chronic colitis

**Figure 1:**
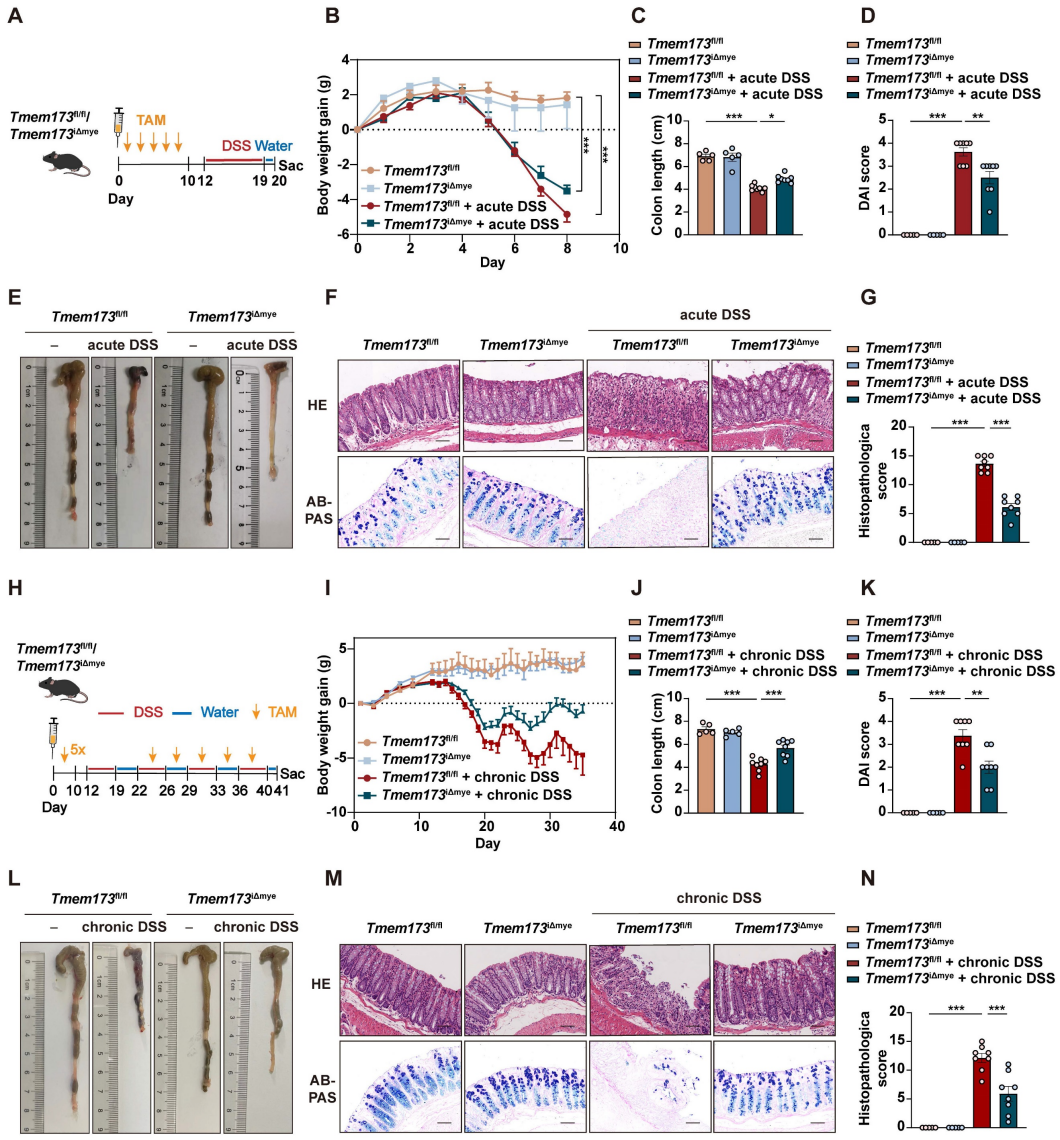
** Myeloid-specific knockout of STING in adult mice ameliorates DSS-induced acute and chronic colitis. (A-G)** *Tmem173*^fl/fl^ mice and* Tmem173*^iΔmye^ mice were induced by acute DSS colitis after tamoxifen (TAM) treatment. **(A)** Animal experimental design. **(B)** Body weight gain. **(C)** Colon length. **(D)** DAI score. **(E)** Representative colon pictures. **(F)** Representative H&E and AB-PAS staining of colonic sections. **(G)** Histopathological score. **(H-N)** *Tmem173*^fl/fl^ mice and* Tmem173*^iΔmye^ mice were induced by chronic DSS colitis after TAM treatment. **(H)** Animal experimental design. **(I)** Body weight gain. **(J)** Colon length. **(K)** DAI score. **(L)** Representative colon pictures. **(M)** Representative H&E and AB-PAS staining of colonic sections. **(N)** Histopathological score. Scale bars, 100 μm. Values represent the mean ± S.E.M. of at least five mice in each group. Statistical significance: *p < 0.05, **p < 0.01, ***p < 0.001.

To define the role of STING pathway in the development of colitis, we utilized C57BL/6N mice to establish DSS-induced acute and chronic colitis models** (Figure S1A and D)**. The phosphorylation of STING and activation of downstream transcription factors, including interferon regulatory factor 3 (IRF3), nuclear factor kappa-B (NF-κB), and IRF7, were all significantly elevated in colitis **(Figure S1B, C, E and F)**. Our results further suggested that global knockout (KO) of STING remarkably inhibited the expression of inflammatory genes and ameliorated colitis, which support the findings of Shmuel-Galia *et.al* 6 but not Yang* et.al* 9 **(Figure S1G-N)**. Since myeloid cell-mediated innate immunity is a master regulator in colitis, we established a tamoxifen (TAM) inducible CreERT2 dependent and myeloid cell-specific STING KO mice (*Tmem173*^iΔmye^) to explore the exact pathogenic role of STING activation. Five continuous times of TAM administration could significantly induce the deletion of STING in adult *Tmem173*^iΔmye^ mice. As shown in **Figure 1A**-**B**, myeloid knockout of STING significantly relieved body weight loss, increased colon length (**Figure 1C-E**), decreased the disease activity index (DAI) score (**Figure 1D**), and reduced spleen coefficient (**Figure S2A**) in acute colitis. Histological analysis further revealed that intestinal epithelial injury, associated with lymphocyte infiltration and mucosal barrier impairment, were dramatically improved in *Tmem173*^iΔmye^ mice when compared with litter-mate *Tmem173*^fl/fl^ mice (**Figure 1F-G**). *Tmem173*^iΔmye^ mice were also significantly protected from prolonged and repeated DSS-induced chronic colitis, as illustrated in **Figure 1H-L**, and **Figure S2B** and showed improved survival rate (**Figure S2C**). Histopathological evaluation further supported above findings (**Figure 1M-N**). From the perspective of cytokine expression, qPCR analysis demonstrated that the upregulation of* Il1b*, *Tnfa*, and *Il6* were diminished in *Tmem173*^iΔmye^ mice in both acute and chronic colitis (**Figure S2D-E**).

**Figure 2:**
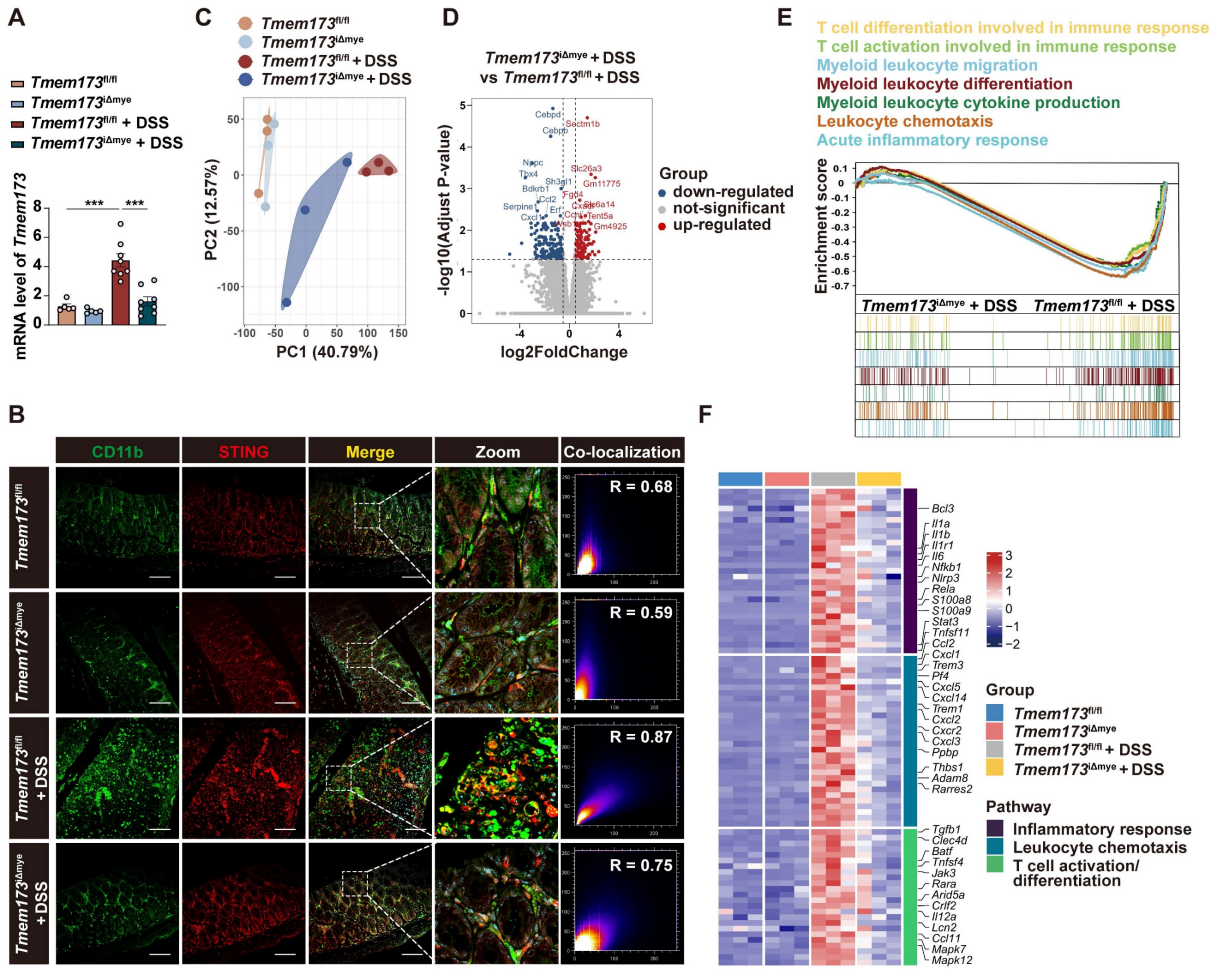
** Effects of myeloid-specific knockout of STING on innate immunity and adaptive immunoregulation. (A)** Relative mRNA levels of *Tmem173* in the colon. **(B)** Representative immunofluorescent co-staining of CD11b and STING of colonic sections. **(C)** The principal components analysis (PCA). **(D)** Volcano plot of DEGs in *Tmem173*^iΔmye^ + DSS *vs Tmem173*^fl/fl^ + DSS. Down-regulated genes are in blue and up-regulated genes are in red. The cutoff values |log2FoldChange| < 0.5 and adjusted p value < 0.05 were utilized to identify differentially expressed genes. **(E)** GSEA analysis on pathways related to innate and adaptive immune responses in *Tmem173*^iΔmye^ + DSS *vs Tmem173*^fl/fl^ + DSS. Scale bars, 100 μm. **(F)** DEGs in pathways related to inflammatory response, leukocyte chemotaxis and T cell activation/differentiation. Values represent the mean ± S.E.M. of at least five mice in each group. Statistical significance: ***p < 0.001.

The myeloid-specific knockout, similar to the systemic knockout of STING, notably decreased the upregulation of STING in inflamed colon tissue (**Figure 2A**), suggesting that in the case of UC, the activation of the STING pathway is primarily in myeloid cells rather than other cell types like epithelial cells. **Figure 2B** further supported this hypothesis. While there is mild increase of STING expression in epithelial cells, a substantial number of infiltrating CD11b and STING double-positive cells are observed in UC (**Figure S2F-J**). In *Tmem173*^iΔmye^ mice, the infiltration of CD11b^+^ cells significantly decreased, along with a specific loss of STING expression in CD11b^+^ cells, leading to an improvement in the structure of the colon barrier.

Previous studies have reported increased susceptibility to colitis in mice with congenital deletion of STING 10. Hence, we are curious to investigate whether the neonatal deletion of myeloid STING actually aggravates colonic inflammation. TAM was intraperitoneally injected into newborn *Tmem173*^fl/fl^ mice and *Tmem173*^fl/fl^*Lyz2-Cre*^ert2^ mice for continuous five days. The administration of DSS was carried out when these mice reached the age of 7 weeks (referred as N*-Tmem173*^fl/fl^ + DSS group and N*-Tmem173*^iΔmye^ + DSS group, respectively) (**Figure S3A**). Interestingly, N-*Tmem173*^iΔmye^ mice exhibited greater body weight loss, colon shortening and disease severity in response to DSS treatment (**Figure S3B-E**). Histological analysis revealed more severe inflammatory infiltration and tissue damage (**Figure S3F-G**). The transcript levels of inflammatory genes, including *Tnfa*, *Il1b*, and *Il6,* were significantly higher in DSS-treated N-*Tmem173*^iΔmye^ mice as well (**Figure S3H**). Contrary to the more severe colitis, there was a decrease in the infiltration of CD11b^+^ myeloid cells and a significant reduction in the expression of STING in the colon of N-*Tmem173*^iΔmye^ mice (**Figure S3I**). This finding suggests that the absence of STING in neonatal mice may disrupt the establishment of immune tolerance in the intestinal lamina propria, warranting further investigation.

### Decreased myeloid-derived dendritic cells and diminished activation of Th1 and Th17 Cells are observed in colitic *Tmem173*^iΔmye^ mice

To investigate the immunoregulatory mechanisms underlying the alleviation of colitis following myeloid-specific STING knockout, we performed RNA-seq analysis. Principal component analysis (PCA) revealed significant differences in the gene expression profile between colitic *Tmem173*^iΔmye^ mice and *Tmem173*^fl/fl^ mice (**Figure 2C**). A total of 2008 DEGs were commonly identified in both *Tmem173*^fl/fl^ + DSS vs. *Tmem173*^fl/fl^ and *Tmem173*^iΔmye^ + DSS vs. *Tmem173*^fl/fl^ + DSS (**Figure S4A**), which are highly likely to be associated with the protective effects of myeloid STING KO. Specifically, 176 downregulated DEGs, including *Cebpd*, *Cebpb*, *Ccl2,* and *Cxcl1*, and 133 upregulated DEGs, including *Sectm1b*, *Slc26a3*, *Fgd4,* and *Slc6a14, were found in Tmem173*^iΔmye^ + DSS vs. *Tmem173*^fl/fl^ + DSS (**Figure 2D**). GO, KEGG and GSEA analysis suggested that these DEGs are enriched in multiple inflammation-related pathways, such as myeloid leukocyte migration, differentiation, and cytokines production, acute inflammatory response, and T cell differentiation and activation involved in immune response (**Figure S4B** and **Figure 2E**). As depicted in **Figure 2F**, downregulation of acute phase response-related genes, such as *S100a8* and *S100a9*, and canonical pro-inflammatory cytokines and chemokines, such as *Il6*, *Il1b*, and *Ccl2,* was along with suppressed expression of genes contributes to leukocyte chemotaxis and activation like *Trem1*, *Cxcl10*, *Il12a*, thereby linking altered innate immunity to adaptive immunoregulation following myeloid STING KO.

**Figure 3:**
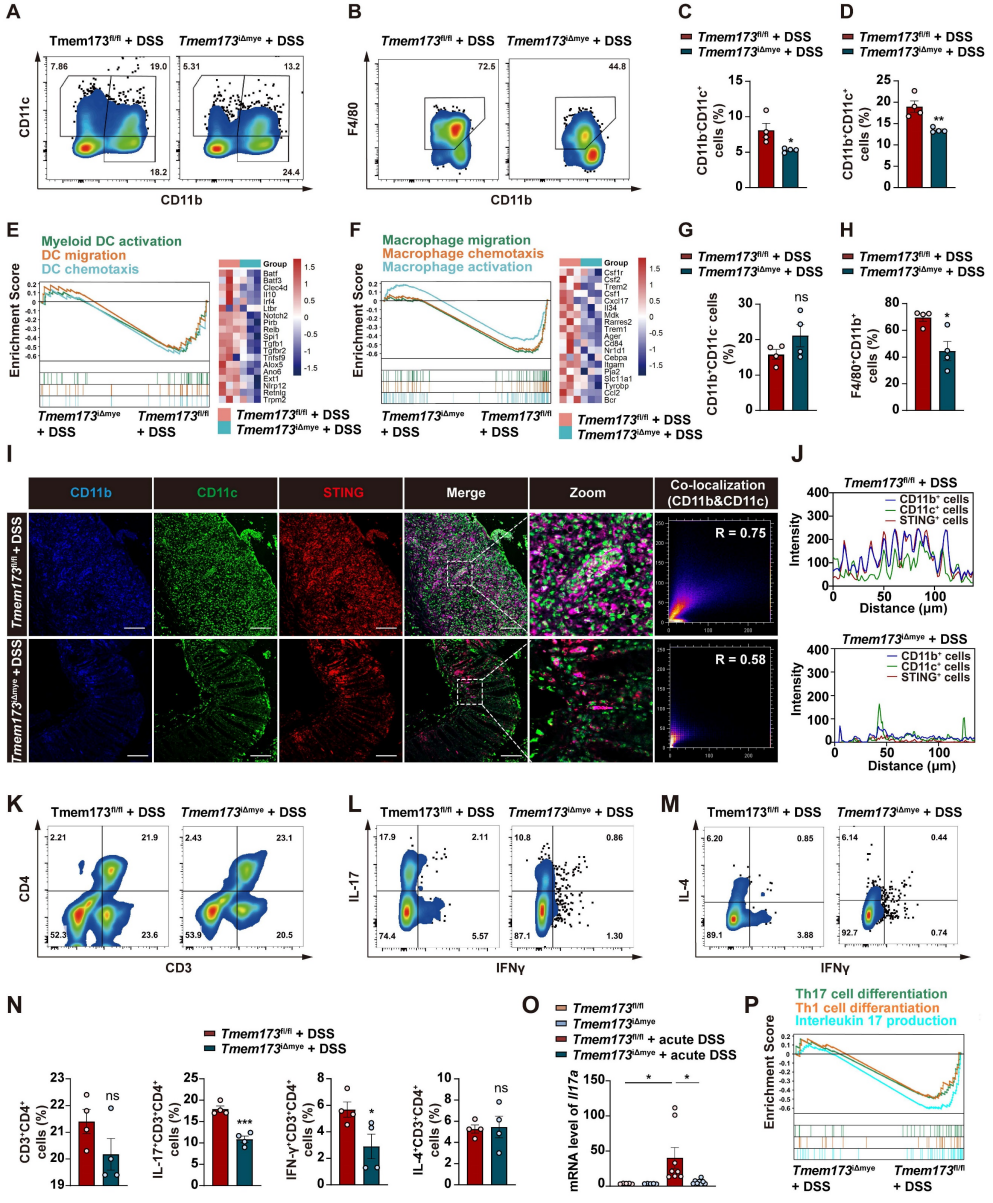
** Effects of myeloid cell-specific STING deletion on DC and macrophage infiltration and Th1/Th17 cell activation in colitis. (A-D, G-H)** Representative flow cytometry results and quantitative analysis of CD11b^-^CD11c^+^ DCs, CD11b^+^CD11c^+^ DCs, CD11b^+^CD11c^-^ monocytes and F4/80^+^CD11b^+^ macrophages in the colonic lamina propria. **(E)** GSEA analysis on pathways related to DC activation, migration, and chemotaxis in *Tmem173*^iΔmye^ + DSS *vs Tmem173*^fl/fl^ + DSS. The relative expression of genes in the leading-edge subset of GSEA analysis is shown alongside as a heatmap. **(F)** GSEA analysis of pathways related to macrophage activation, migration, and chemotaxis in *Tmem173*^iΔmye^ + DSS *vs Tmem173*^fl/fl^ + DSS and heatmap. **(I-J)** Representative immunofluorescence co-staining of CD11b, CD11c and STING in the colon and the quantitative analysis. The co-localization of CD11b and CD11c in *Tmem173*^fl/fl^ + DSS group and *Tmem173*^iΔmye^ +DSS group were calculated with overlap coefficient R. **(K-N)** Representative flow cytometry results and quantitative analysis of CD3^+^CD4^+^ T cells, IL-17^+^ CD3^+^CD4^+^ Th17 cells, IFNγ^+^CD3^+^CD4^+^ Th1 cells, and IL-4^+^CD3^+^CD4^+^ Th2 cells in the colonic lamina propria. **(O)** Relative mRNA levels of *Il17a* in the colon. **(P)** GSEA analysis on pathways related to Th1 and Th17 differentiation, and IL-17 production in *Tmem173*^iΔmye^ + DSS *vs Tmem173*^fl/fl^ + DSS. Scale bars, 100 μm. Values represent the mean ± S.E.M. of at least four mice in each group. Statistical significance: ns, not significant, *p < 0.05, **p < 0.01, ***p < 0.001.

To further investigate the potential connection between innate and adaptive immunity, we utilized Cibersort to analyze the proportion of various immune cells based on RNA-seq data. (**Figure S5A-B**). Specifically, increased abundance of monocytes, immature DCs, activated DCs, M0 macrophage, Th1 and Th17 cells was observed in the colon from colitic *Tmem173*^fl/fl^ mice, which was all decreased to a certain extent in *Tmem173*^iΔmye^ mice (**Figure S5C**). We thus hypothesized that the absence of STING in myeloid cells alleviated intestinal inflammation by reducing DC and macrophage infiltration, and Th1 and Th17 cell differentiation. To test the hypothesis, we evaluated immune cell profiles in the lamina propria cells isolated from colon of colitic *Tmem173*^fl/fl^ mice and colitic* Tmem173*^iΔmye^ mice using flow cytometry. The immune cell sorting strategy was shown in the **Figure S6A**. As depicted in **Figure S6B-C,** the infiltration of CD45^+^ cells from colonic lamina propria was significantly decreased in colitic* Tmem173*^iΔmye^ mice. Myeloid STING deletion significantly reduced the frequency of CD11b^-^CD11c^+^ DCs and CD11b^+^CD11c^+^ DCs in response to DSS treatment (**Figure 3A, C-D**), while CD11b^+^CD11c^-^ myeloid cells displayed moderate increase in colitic* Tmem173*^iΔmye^ mice without statistical significance (**Figure 3A and G**). These findings are consistent with RNA-seq analysis (**Figure 3E**). Although CD11b^+^CD11c^-^ myeloid cells were accumulated in *Tmem173*^iΔmye^ mice, a significant reduction of F4/80^+^CD11b^+^CD11c^-^ macrophages was observed (**Figure 3B and H**), suggesting abrogated maturation of macrophages. GSEA analysis and corresponding gene expressions further revealed that myeloid cell-specific STING deletion negatively regulated signaling pathways involved in macrophage migration, chemotaxis, and activation (**Figure 3F**). The potential deficiency in GM-CSF- and M-CSF-mediated macrophage differentiation (**Figure S7A**) in adult *Tmem173*^iΔmye^ mice subjected to DSS treatment suggests a possible connection between myeloid STING and macrophage maturation. This deficiency may contribute to the disruption of immune tolerance in neonatal myeloid STING KO mice. In DSS-induced chronic colitis, similar results were found (**Figure S7B-D**). In addition to support the results of cytometry, IF analysis further demonstrated that STING activation was predominantly found in significantly infiltrated CD11b^+^CD11c^+^ and CD11b^+^CD11c^-^, but not in CD11b^-^ cells in *Tmem173*^fl/fl^ mice. Notably, both the infiltration of CD11b^+^ cells and the colocalization of STING with CD11b^+^ cells were diminished in *Tmem173*^iΔmye^ mice (**Figure 3I-J**).

Myeloid-derived DCs have long been characterized as vital mediators of the recruitment and activation of pro-inflammatory helper T cells, including Th1, Th2, and Th17 cells 12, 13. As expected, along with a decreased number of CD3^+^CD4^+^ T cells in colitic *Tmem173*^iΔmye^ mice (**Figure 3K and N**), the frequencies of IFN-γ^+^ Th1 and IL-17^+^ Th17 cells in the lamina propria were significantly reduced (**Figure 3L and N**). However, the proportion of IL-4^+^ Th2 cell remained unchanged (**Figure 3M-N**). Among these findings, the reduction of Th17 phenotype was most significant and was further supported by qPCR analysis (**Figure 3O**). A similar downregulation of *Il17a* was also seen in chronic colitis (**Figure S7E**). GSEA analysis also indicated that T cell-related pathways, including Th1 and Th17 cell differentiation, and IL-17 production were all inhibited in *Tmem173*^iΔmye^ mice in response to DSS, when compared to *Tmem173*^fl/fl^ mice (**Figure 3P**). Therefore, myeloid cell-specific STING deletion attenuated intestinal inflammation through suppressing DC and macrophage infiltration and Th1/Th17 cell activation in colon.

### T helper cell priming IL-12 family cytokines are predominantly produced by DCs in response to STING-TBK1-IRF3/NF-κB activation in colitis

**Figure 4:**
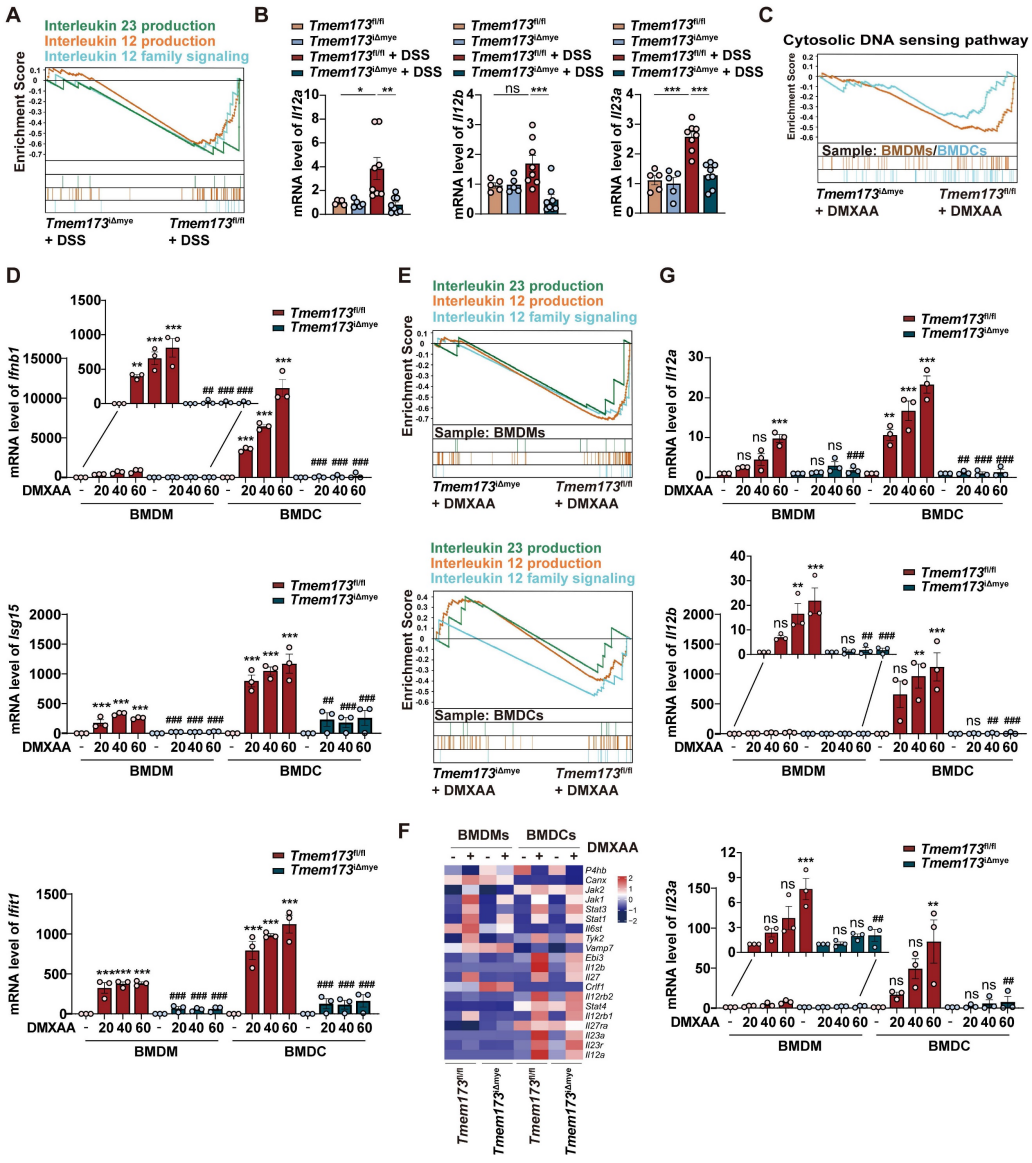
** Myeloid-specific knockout of STING inhibits IL12/IL23 expression *in vivo* and *in vitro*. (A)** GSEA analysis of IL-12 family pathways in *Tmem173*^iΔmye^ + DSS *vs Tmem173*^fl/fl^ + DSS. **(B)** Relative mRNA levels of *Il12a*, *Il12b*, and *Il23a* in the colon. Values represent the mean ± S.E.M. of at least five mice in each group. Statistical significance: ns, no significant, *p < 0.05, **p < 0.01, ***p < 0.001. **(C-G)** WT and STING KO BMDMs and BMDCs were treated with DMXAA (DX). **(C)** GSEA analysis of cytosolic DNA sensing pathway in *Tmem173*^iΔmye^ + DMXAA *vs Tmem173*^fl/fl^ + DMXAA in BMDMs and BMDCs. **(D)** Relative mRNA levels of *Ifnb1*, *Isg15*, and *Ifit1*. **(E)** GSEA analysis of IL-12 family pathways in *Tmem173*^iΔmye^ + DMXAA *vs Tmem173*^fl/fl^ + DMXAA in BMDMs and BMDCs, and **(F)** the relative expression of genes in the leading-edge subset. **(G)** Relative mRNA levels of *Il12a*, *Il12b*, and *Il23a* in primary BMDMs and BMDCs. Values represent the mean ± S.E.M. of at least three samples in each group. Statistical significance relative to *Tmem173*^fl/fl^ group: ns, no significant, *p < 0.05, **p < 0.01, ***p < 0.001. Statistical significance relative to *Tmem173*^fl/fl^ + DMXAA group: ns, not significant, ^##^p < 0.01, ^###^p < 0.001.

Macrophages and DCs are known to be pivotal in priming and activating Th1 and Th17 cells through IL-12 and IL-23 secretion 13-15. As shown in **Figure 4A**, GSEA analysis indicated that in acute colitis model, IL-12/23 production and IL-12 signaling pathways were enriched in *Tmem173*^fl/fl^ mice, which was verified by qPCR results (**Figure 4B**). The reduction of *Il12a*, *Il12b*, and *Il23a* expression was also observed in chronic colitis (**Figure S7F**). To further investigate whether the activation of IL-12 signaling in colitis is dependent on STING, bone marrow-derived macrophages (BMDMs) and bone marrow-derived dendritic cells (BMDCs) isolated from* Tmem173*^fl/fl^ mice and *Tmem173*^iΔmye^ mice were treated with STING agonist Vadimezan (DMXAA) and RNA-seq analysis was applied. Based on RNA-seq analysis, both BMDCs and BMDMs exhibit high expression of their respective cell-specific marker genes, confirming the purity and distinction of BMDCs and BMDMs (**Figure S8A**). As speculated, the activation of STING pathway (**Figure 4C**), inflammation-related pathways, and pathways involved in the maturation and activation of macrophages and DCs (**Figure S8B-D**) were significantly stimulated by DMXAA in both BMDMs and BMDCs derived from *Tmem173*^fl/fl^ mice. However, these effects were reversed in BMDMs and BMDCs derived from *Tmem173*^iΔmye^ mice. Consistently, qPCR results indicated that the mRNA expression of interferon (IFN) and IFN-stimulated genes (ISGs) were significantly decreased in both STING KO BMDMs and BMDCs after DMXAA treatment (**Figure 4D**). Likewise, in RNA-seq results, IL-12 and IL-23 production-related pathways were enriched in DMXAA-treated BMDMs and BMDCs derived from *Tmem173*^fl/fl^ mice, but not STING KO cells (**Figure 4E-F**). Notably, DMXAA induced higher expression of STING downstream genes and IL-12 family cytokines in BMDCs, when compared with BMDMs. Especially, *Il12a*, *Il12b* and *Il23a* were predominantly expressed in BMDCs instead of BMDMs (**Figure 4G**). The different capacities in producing IL-12 family cytokines between BMDCs and BMDMs suggested that the activation of T helper cells in colitis were largely dependent on STING activation in myeloid-derived DCs.

**Figure 5:**
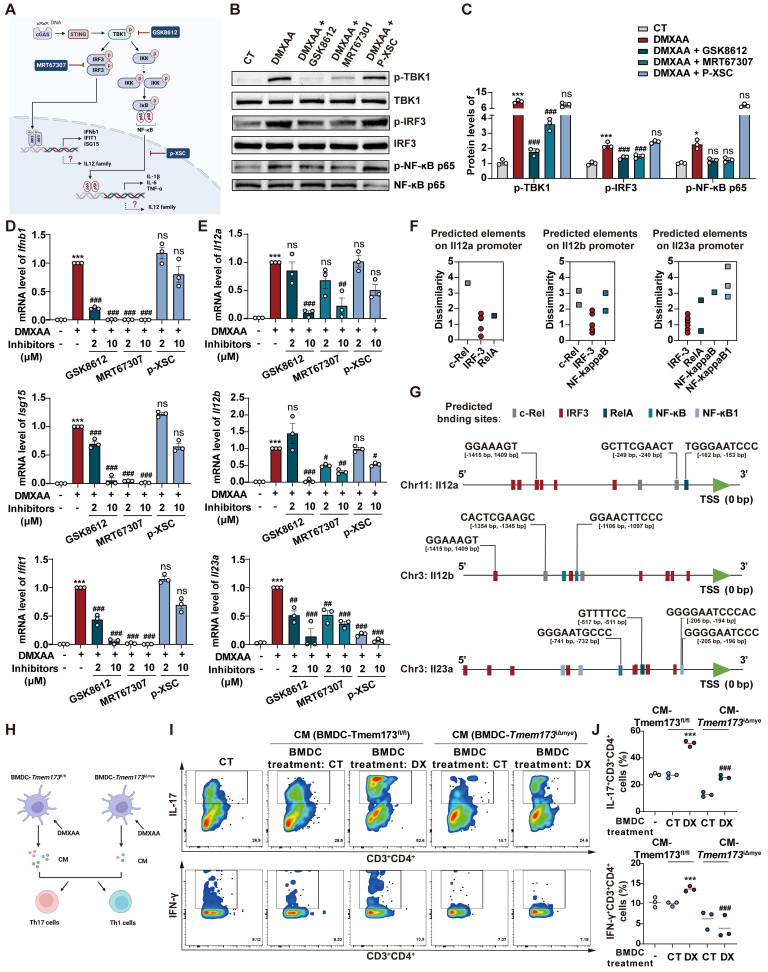
** IRF3 and NF-κB are indispensable in the STING activation-mediated transcription of IL-12 family cytokines. (A)** The Illustration of the downstream signaling cascades of STING and specific inhibitors. **(B-E)** Primary WT BMDCs were stimulated with 40 μg/mL DMXAA to activate STING pathway, with or without the treatment of GSK8612, MRT6730, and p-XSC for 8 h. **(B-C)** Representative images and quantitative analysis of immunoblotting detecting phosphorylation of TBK1, IRF3, and NF-κB p65. **(D)** Relative mRNA levels of *Ifnb1*, *Isg15*, and *Ifit1*. **(E)** Relative mRNA levels of *Il12a*, *Il12b*, and *Il23a*. Values represent the mean ± S.E.M. of at least three samples in each group. Statistical significance relative to vehicle control: *p < 0.05, ***p < 0.001. Statistical significance relative to DMXAA group: ns, not significant, ^#^p < 0.05, ^##^p < 0.01, ^###^p < 0.001. **(F)** The number and dissimilarity of predicted elements of IRF3 and NF-κB on the promoters of IL-12 family genes. **(G)** The distribution of predicted binding sites of IRF3 and NF-κB on promoters of IL-12 family cytokines. Colored boxes represent transcription factor binding sites. **(H)** Primary WT and STING KO BMDCs were stimulated with 40 μg/mL DMXAA for 8 h and the conditioned medium (CM) was collected. Differentiated splenic Th1 and Th17 cells were incubated with BMDCs-derived CM for 36 h before cells were collected for flow cytometry. **(I-J)** Representative flow cytometry results and quantitative analysis of splenic IL-17^+^ CD3^+^CD4^+^ Th17 cells and IFNγ^+^CD3^+^CD4^+^ Th1 cells. Values represent the mean ± S.E.M. of at least three samples in each group. Statistical significance relative to CM(*Tmem173*^fl/fl^-CT) group: ***p < 0.001. Statistical significance relative to CM(*Tmem173*^fl/fl^-DX) group: ^###^p < 0.001.

However, critical evidence is still lacking regarding whether IL-12 family cytokines are direct downstream targets of STING activation. As anticipated, DMXAA remarkably induced the activation of TANK-binding kinase 1 (TBK1), IRF3, and NF-κB, and the degradation of inhibitory subunit of nuclear factor kappa-B alpha (IκBα) in BMDCs (**Figure S9A**). In **Figure 5A**, TBK1 inhibitor GSK8612, IRF3 inhibitor MRT67307, and NF-κB inhibitor p-XSC were then used to elucidate the specific downstream transcription factors and signaling pathways involved in STING-mediated IL-12 expression. The effects of these inhibitors were shown in **Figure 5B-C**. GSK8612 and MRT67307 effectively inhibited the phosphorylation of TBK1 and downstream IRF3, respectively, also both slightly suppressed NF-κB activation in DMXAA-induced BMDCs. As the negative regulator of the binding of NF-κB and DNA, P-XSC had no significant influence on TBK1/IRF3 pathway and NF-κB phosphorylation.

Furthermore, the expressions of type I IFN and ISGs in response to STING activation were significantly abrogated by TBK1 and IRF3 inhibitors, rather than NF-κB inhibitor, which were complied with previous reports (**Figure 5D**). However, all inhibitors attenuated the DMXAA-induced expression of IL-12 family genes, albeit with varying potencies. Interestingly, MRT67307 exhibited greater suppressive effects on *Il12a* and *Il12b* expression than p-XSC, whereas P-XSC displayed more pronounced restriction on the transcription of *Il23a* (**Figure 5E**). These effects are potentially attribute to the presence of more binding elements of IRF3 on the promoter regions of *Il12a* and *Il12b*, as well as more NF-κB binding sites on the *Il23a* promoter (**Figure 5F-G**, and **Figure S9B-D**). Functioned as a specific inhibitor of TBK1, which is the shared upstream factor of IRF3 and NF-κB, GSK8612 consistently inhibited the expressions of all IL-12 family genes (**Figure 5E**). These findings indicated that both IRF3 and NF-κB are indispensable in the STING activation-mediated transcription of IL-12 family cytokines.

The effects of IL-12 family cytokines derived from DMXAA-treated WT and STING KO BMDCs on Th1 and Th17 differentiation of murine splenic T cells were further confirmed (**Figure 5H**). In the process of differentiating splenetic CD3^+^CD4^+^ T cells, the proportion of IL-17^+^ Th17 cells and IFNγ^+^ Th1 cells were found to be significantly higher when exposed to conditioned medium derived from DMXAA-treated WT BMDCs. However, these proportions decreased when exposed to conditioned medium derived from DMXAA-treated STING KO BMDCs (**Figure 5I-J**). GSEA analysis based on RNA-seq results further suggested that upregulated DEGs associated with Th1 and Th17 differentiation, IL-17 signaling, antigen-processing and presentation were all enriched in DMXAA-treated WT BMDCs, when compared with STING KO mice (**Figure S9E**). These findings demonstrate that IL-12 family cytokines are predominantly produced by DCs and favor Th1 and Th17 cell priming.

### TFAM-associated mtDNA derived from damaged intestinal epithelial cells activates STING signaling in BMDCs to promote IL-12 signaling

**Figure 6:**
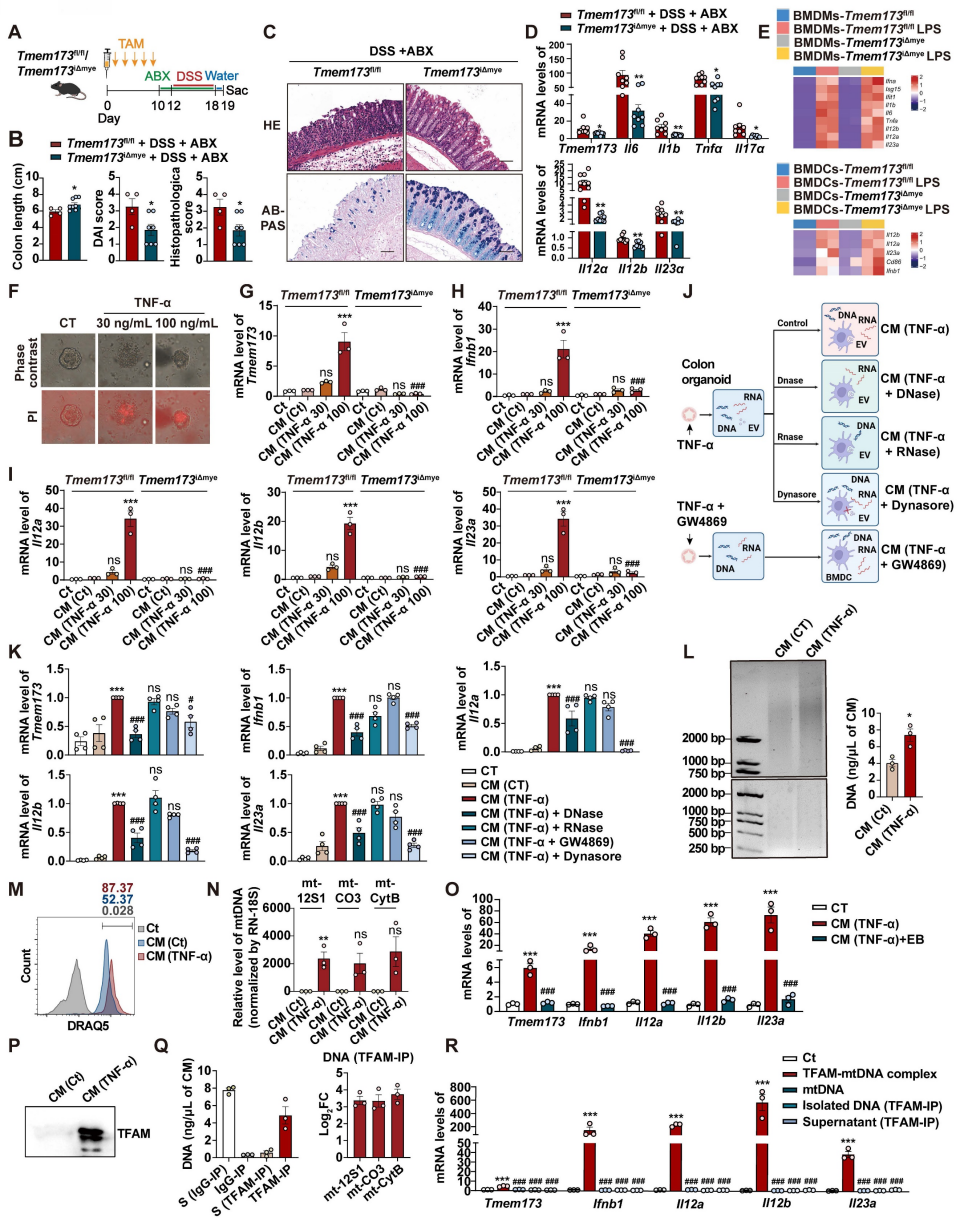
** TFAM-associated mtDNA derived from damaged colonic organoids activates STING signaling in BMDCs to promote IL12 signaling.** *Tmem173*^fl/fl^ mice and *Tmem173*^iΔmye^ mice were treated by antibiotic cocktail (ABX) and then subjected to acute DSS administration. **(A)** Animal experimental design. **(B)** Colon length, DAI score, and histopathological score. **(C)** Representative H&E and AB-PAS staining of colonic sections. Scale bars, 100 μm.** (D)** Relative mRNA levels of *Tmem173*, *Il6*, *Il1b*, *Tnfa*, *Il17a*, *Il12a*, *Il12b*, and *Il23a* in the colon. Values represent the mean ± S.E.M. of at least three samples in each group. Statistical significance: *p < 0.05, **p < 0.01. **(E)** Primary WT and STING KO BMDMs and BMDCs were stimulated with LPS for 8 h. Relative mRNA levels of genes of type I IFNs and IL-12 family are shown as a heatmap.** (F-I)** The colonic organoids were treated with vehicle control or TNF-α (30 ng/mL or 100 ng/mL) and the conditioned medium (CM) was collected.** (F)** Representative images of PI staining of colonic organoids. **(G-I)** WT and STING KO BMDCs were incubated with indicated CM and the relative mRNA levels of *Tmem173*, *Ifnb1*, *Il12a*, *Il12b*, and *Il23a* gene are shown. **(J-N)** The colonic organoids were treated with vehicle control, 100 ng/mL TNF-α, or co-treated with 100 ng/mL TNF-α and 10 μM GW4869, and the CM was collected, named as CM (CT), CM (TNF-α), and CM (TNF-α+GW4869), respectively. Some CM samples were further treated by DNase (100 U/mL) and RNase (10 μg/mL), named as CM (TNF-α) + DNase and CM (TNF-α) + RNase. WT BMDCs were incubated with indicated CM with or without 80 μM Dynasore. **(K)** Relative mRNA levels of *Tmem173*, *Ifnb1*, *Il12a*, *Il12b*, and *Il23a*. **(L)** Representative image of DNA gel detecting free DNA fragments in the indicated CM. DNA concentrations are shown alongside. **(M)** The indicated CM was incubated with 5 mM DRAQ5 DNA probe. Representative flow cytometry results and quantitative analysis of DRAQ5-labeled DNA uptake in BMDCs.** (N)** The DNA levels of *mt-12S1*, *mt-CO3*, *mt-CytB*, were normalized by nuclear gene *RN-18S*. **(O)** WT BMDCs were incubated with CM (TNF-α) and EtBr-treated CM (TNF-α). Relative mRNA levels of *Tmem173*, *Ifnb1*, *Il12a*, *Il12b*, and *Il23a* are shown. **(P)** Representative image of immunoblotting detecting TFAM in the CM. **(Q)** DNA quantifications in the supernatant or precipitate from the IP experiment using IgG antibody-coated beads or anti-TFAM antibody-coated beads. The log_2_FC values of *mt-12S1*, *mt-CO3*, and *mt-CytB* in immunoprecipitated TFAM-DNA complex are shown alongside. S (Supernatant).** (R)** WT BMDCs were incubated with TFAM-mtDNA complex, purified free mtDNA (mtDNA), free DNA isolated from TFAM-DNA complex (isolated DNA(TFAM-IP)), or the supernatant from TFAM-IP experiment (supernatant (TFAM-IP)). Relative mRNA levels of *Tmem173*, *Ifnb1*, *Il12a*, *Il12b*, and *Il23a* are shown. Values represent the mean ± S.E.M. of at least three samples in each group. Statistical significance relative to Ct or CM (Ct) groups: ***p < 0.001; relative to CM (TNF-α) or TFAM-mtDNA complex groups: ^###^p < 0.001.

In light of a recent study, we were motivated to explore whether the activation of colitis-associated myeloid STING is triggered by the intestinal microbiota 6. We first treated mice with antibiotic cocktail (ABX) for three days before DSS administration to eliminate intestinal flora (**Figure S10A**). ABX treatment had no influence on DSS-induced elevated phosphorylation levels of STING and downstream transcription factors, including IRF3, NF-κB and IRF7 (**Figure S10B-C**). As depicted in **Figure 6A-C, and S10D**, myeloid knockout of STING still effectively attenuated DSS-induced acute colitis even though the mice were given ABX, as illustrated by elevated body weight, increased colon length, relieved disease activity, and improved colonic epithelium damage. The reduction mRNA levels of *Teme173*, pro-inflammatory cytokines and IL-12 family genes were also found in *Tmem173*^iΔmye^ + DSS + ABX group when compare with *Tmem173*^fl/fl^ + DSS + ABX group (**Figure 6D**). Besides, in the treatment of DSS plus ABX, the activation of STING and downstream transcription factors, including IRF3, NF-κB and IRF7, were all significantly reduced in *Tmem173*^iΔmye^ mice (**Figure S10E-F**). Since Toll-like receptors (TLRs) are the predominant sensor of microbiota-derived PAMPs and are also upstream adaptor protein of type I IFN, we treat WT and STING KO BMDMs and BMDCs with lipopolysaccharide (LPS). The deletion of STING failed to reverse the induction of type I IFN-related genes and IL-12 family genes upon LPS stimulation in BMDMs (**Figure 6E**). Likewise, the expression of *Ifnb1*, IL-12 family genes and DC marker *Cd86* still obviously increased in STING-deficient BMDCs in response to LPS (**Figure 6E**). The deletion of STING also failed to reverse the immune activation induced by other bacterial components, such as MDP (NLRs activator), peptidoglycan (TLR2 activator) and flagellin (TLR5 activator) in BMDMs and BMDCs (**Figure S10G**). The co-localization of STING, CD11b and CD11c and the numbers of CD11b^+^CD11c^+^ cells further supported above findings (**Figure S10G-H**). These data demonstrated that bacteria and bacteria-produced PAMPs are not involved in either the pathogenic roles of STING activation in colonic inflammation or the protective effects of STING deficiency against colitis.

We then hypothesized that DAMPs activated myeloid STING in response to intestinal epithelial damage. Based on the above results showing that DCs, rather than macrophages, predominantly produce IL-12 family cytokines in colitis, we conducted following experiments using BMDCs instead of BMDMs. As shown in **Figure 6F**, treatment with 100 ng/mL TNF-α resulted in significant injuries in primary colonic organoids. These injuries were characterized by PI permeabilization and epithelial destruction, which mimicked the damage observed in colitis-induced intestinal epithelial damage. The conditioned medium of damaged colonic organoids dramatically induced the expression of *Tmem173*, *Ifnb1*, *Il12a*, *Il12b,* and *Il23a* in WT BMDCs. However, these effects were almost totally blunted in STING KO BMDCs (**Figure 6G-I**).

**Figure 7:**
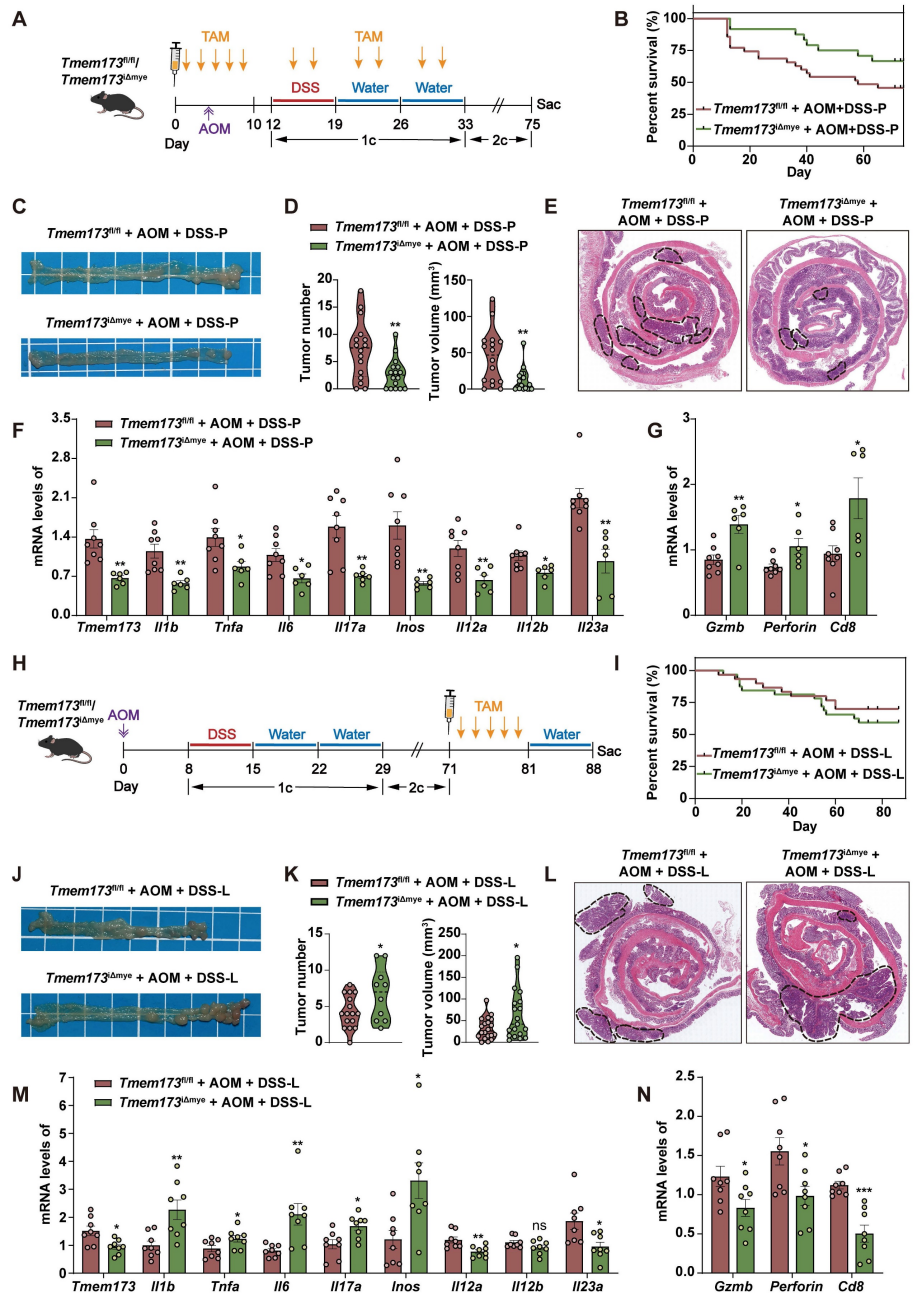
** Inducing myeloid STING knockout at different time points has opposite effects on the progression of colitis-associated cancer. (A-G)** TAM administration was given before the onset of inflammation in *Tmem173*^fl/fl^ mice and* Tmem173*^iΔmye^ mice in the AOM/DSS-induced CAC model. **(A)** Animal experimental design. **(B)** Survival rate. **(C)** Representative pictures of colon samples. **(D)** Tumor number and volume. **(E)** Representative H&E staining of colonic sections. **(F)** Relative mRNA levels of *Tmem173*, *Il1b*, *Tnfa*, *Il6*, *Il17a*, *Inos*, *Il12a*, *Il12b*, and *Il23a* in the normal colon tissues adjacent to tumors. **(G)** Relative mRNA levels of *Gzmb*, *Perforin*, *Cd8* in the tumors. **(H-N)** TAM administration was given after the formation of tumor in *Tmem173*^fl/fl^ mice and* Tmem173*^iΔmye^ mice in the AOM/DSS-induced CAC model. **(H)** Animal experimental flowchart. **(I)** Survival rate. **(J)** Representative colon pictures. **(K)** Tumor number and volume. **(L)** Representative H&E staining of colonic sections. **(M)** Relative mRNA levels of *Tmem173*, *Il1b*, *Tnfa*, *Il6*, *Il17a*, *Inos*, *Il12a*, *Il12b*, and *Il23a* in the normal colon tissues adjacent to tumors. **(N)** Relative mRNA levels of *Gzmb*, *Perforin*, *Cd8* in tumors. Values represent the mean ± S.E.M. of at least five mice in each group. Statistical significance: ns, no significant, *p < 0.05, **p < 0.01, ***p < 0.001.

To investigate whether DAMPs were transported *via* extracellular vesicles (EVs) system as reported previously 11, colonic organoids were pre-treated with GW4869 to inhibit the release of damage-associated EVs. Conditioned medium was also incubated with DNase and RNase to remove cell free DNA and RNA, respectively. BMDCs were co-treated with Dynasore to restrict endocytosis of either damage-associated EVs or cell free nucleotide (**Figure 6J**). Collectively, as depicted in **Figure 7F**, compared to CM (TNF-α) group, only CM (TNF-α) +DNase group and CM (TNF-α) +Dynasore group exhibited significant lower mRNA levels of *Tmem173*, *Ifnb1*, *Il12a*, *Il12b,* and *Il23a*.This suggests that the uptake of damage-associated cell free DNA through endocytosis, but not RNA or EVs, played a crucial role in activating the STING pathway in BMDCs, leading to the induction of IL-12 family gene expression (**Figure 6K**).

As anticipated, cell-free DNA fragments were released from TNF-α-damaged colonic organoids into the conditioned medium (**Figure 6L**), and were then uptake by BMDCs, as illustrated by the presence of DRAQ5-labeled damage-associated DNA fragments (**Figure 6M**). To determine the source of these DNAs, qPCR was performed to examine mitochondrial genes *mt-12S1*, *mt-Co3*, and *mt-Cytb* and nuclear gene *RN-18S*. The results showed that mtDNAs instead of nuclear DNAs were enriched in the conditioned medium derived from damaged organoids (**Figure 6N**). The removal of mtDNA by Ethidium Bromide (EtBr), a commonly used mtDNA specific eliminator, significantly blunted the expression of *Tmem173*, *Ifnb1*, *Il12a*, *Il12b,* and *Il23a* in BMDCs induced by the CM-derived damaged colonic organoid (**Figure 6O**). Notably, the protein level of mitochondrial nucleoid organizing protein TFAM was also increased in the conditioned medium (**Figure 6P**), which has been reported to enhance the detection of mtDNA by cGAS through DNA prearrangement 16. Furthermore, through IP using a TFAM-specific antibody, we found that most of the DNA fragments in the conditioned medium were associated with TFAM and identified as mtDNA (**Figure 6Q**). The immunoprecipitated TFAM-mtDNA complex, free mtDNA isolated from the TFAM-IP precipitate, and the supernatant of TFAM-IP were prepared based on the same volume of CM (TNF-α) used in other experiments in **Figure 6**, and were used to stimulate BMDC. As shown in **Figure 6R**, only TFAM-mtDNA complex from CM (TNF-α) could activate BMDC, while other treatments, including purified mtDNA extracted from liver (about 1.2 ng/μL final concentration as a mtDNA control) failed to trigger responses in BMDCs. These results demonstrated that TFAM-associated, but not free, mtDNA derived from damaged colonic epithelial activated STING and its downstream IL-12 signaling in BMDCs.

### Inducing myeloid STING knockout at different time points has opposite effects on the progression of colitis-associated tumorigenesis

Patients with colitis have substantial risk of developing colitis-associated carcinoma (CAC) 17. To further explore whether myeloid-specific STING knockout at the inflammation phase protects tumorigenesis, we established an AOM/DSS-induced CAC murine model. We intraperitoneally injected TAM into mice prior to DSS induction to ablate myeloid STING before the occurrence of inflammation (referred as *Tmem173*^fl/fl^ AOM+DSS-P group and *Tmem173*^iΔmye^ AOM+DSS-P group) (**Figure 7A**). As anticipated, following the induction of AOM/DSS, there was a notable increase in the survival rate of mice with myeloid ablation of STING compared to *Tmem173*^fl/fl^ mice, while the body weight gain had no significant difference between two groups (**Figure 7B and S11A**). The deficiency of myeloid STING also resulted in significant protection against tumor development, as evidenced by reduced tumor numbers and volumes (**Figure 7C-D**). H&E staining of colon sections supported these findings (**Figure 7E**). The mRNA levels of *Tmem173*, and inflammatory genes associated with colitis progression were all significantly downregulated, while the expression of genes involved in cytotoxic lymphocyte (CTL)-mediated tumor immunity, including *Gzmb*, *Perforin*, and *Cd8* were all significantly elevated in *Tmem173*^iΔmye^ AOM+DSS-P group, when compared with *Tmem173*^fl/fl^ mice **(Figure 7F-G)**. These results demonstrate that early myeloid STING deletion can delay tumor occurrence and hinder tumor favoring cytokine-induced cell proliferation by alleviating excessive colonic inflammation. Importantly, it does not impede the process of anti-tumor immunity; instead, it enhances CTL function.

Inspired by the contrasting disease progression observed in neonatal and adult myeloid STING KO mice, we conducted further investigations to determine if myeloid STING KO after tumor formation would yield different outcomes. We thus performed TAM administration after three circles of AOM/DSS induction (referred as *Tmem173*^fl/fl^ AOM+DSS-L group and *Tmem173*^iΔmye^ AOM+DSS-L group) (**Figure 7H**). There was no significant difference in the number and volume of tumors between these two groups before TAM induction (**Figure S11B-C**). Interestingly, the ablation of myeloid STING after tumor formation in turn lead to more severe tumor growth comparable to *Tmem173*^fl/fl^ mice (**Figure 7I-L** and **Figure S11D**). Cibersort analysis baes on RNA-seq results further suggested an overall “cold” or immunosuppressive tumor environment (**Figure S12A**). The mRNA levels of *Tmem173*, tumor favoring inflammatory genes and IL-12 family genes were all remarkably increased in the tumor of *Tmem173*^iΔmye^ mice (**Figure 7M**), in line with suppressed STING-related type I IFN pathways, and impaired antigen presentation, and macrophage and DC activation (**Figure S12B-D**). T cell chemotaxis and activation, T cell-mediated cytotoxicity and other immune responses to tumor cells were also inhibited by lateral myeloid STING KO after tumor formation (**Figure S11E-F**, and **Figure S12B-D**), which is further supported by qPCR results showing downregulated *Gzmb*, *Perforin*, and *Cd8* (**Figure 7N**)**.** As depicted in **Figure S12E**, both the number of CD8^+^ cells and the co-localization of CD8 and Perforin were remarkably decreased in tumor of *Tmem173*^iΔmye^ AOM+DSS-L group, suggesting a malfunction in CTL-mediated tumor immunity following myeloid STING depletion. The results indicated that deleting STING in myeloid cells before the onset of inflammation alleviated AOM/DSS-induced CAC. Conversely, eliminating STING in myeloid cells after tumor formation enhanced tumor growth by modifying the tumor microenvironment to a more immunologically inactive state.

## Discussion

STING activation in myeloid cells has emerged as a critical immune factor in the pathogenesis of UC 5, 11. In N153S mice with constitutive activation of STING, STING in CD11b^+^ myeloid cells induced severe colonic inflammation through enhancing protein stabilization mediated by K63-linked ubiquitination 6. Besides, Ahn and coworkers previously shown that specific knockout of STING in mononuclear phagocytes, including Lysm^+^ cells and CD11c^+^ cells, inhibited tumor formation in response to AOM/DSS induction 7. In our data, myeloid deletion of STING in adult mice significantly ameliorated DSS-induced acute and chronic colitis. These results all suggested that blockade of myeloid STING may become a potential therapeutic target for the treatment of excessive inflammation in colitis and CAC. Although it was generally believed that the suppression of STING signaling effectively relieved colitis, Canesso *et al.* found that mice with congenital knockout of STING were defective in the development of mucosal barrier and intestinal immune system, which led to ultimate aggravation of colitis 10. Moreover, it was demonstrated that ablation of cGAS exacerbated colitis and CAC due to intestinal stem cell deficiency and impaired intestinal barrier integrity 18. Our results also found that the deletion of myeloid SING in newborn mice exacerbated adult-onset of colonic inflammation. Despite the need for further evidence, we can infer from the accumulation of immature macrophages following myeloid STING knockout in adult mice that the congenital absence of myeloid STING may disrupt the establishment of intestinal immune homeostasis, thereby exacerbating the pathological manifestations of colitis. Notably, Zhang *et al.* recently reported that STING1 localized to the nucleus to activate transcription factor aryl hydrocarbon receptor (AHR) and competitively inhibited IRF3-meidated immune response. This nuclear STING ameliorated DSS-induced colitis by restoring gut microbial homeostasis. This finding offers another possible explanation for the protective effects of STING. However, the authors also noticed that 2'3'-cGAMP possessed higher affinity to STING when compared with the ARH ligands, and can direct AHR ligand-mediated nuclear translocation of STING to ER-ERGIC transportation, favoring canonical IRF3 activation. This suggests that nuclear STING may preferentially maintain intestinal immune tolerance in the homeostatic colon, while in the presence of DAMPs, the cGAS-STING-IRF3 pathway primarily licenses excessive inflammation 19. The paradoxical effects of STING knockout at different stages of disease progression observed in various conditions are also evident in the colitis-associated cancer transformation process. The constraint of STING activity before cancer initiation protects against tumor development, which is consistent with a recent study 7. However, once tumors have formed, STING deletion promotes the generation of an immunosuppressive microenvironment, thereby enhancing tumor growth. These findings emphasize the need for careful consideration of the timing of STING targeting in colitis treatment, based on the intestinal immune environment response and disease progression status, to maximize therapeutic benefits without inadvertently promoting disease progression.

Intestinal DCs are believed to function as crucial initiators and regulator of innate and adaptive immune responses in mucosal barrier. Different subsets of DCs exhibited distinct functions on the severity of colitis in murine models 20. Various studies have demonstrated that CD11b^+^CD11c^+^ DCs accelerated the pathogenesis of colitis by producing massive inflammatory cytokines, such as TNF-α, IL-6, IL-17, IL-12, IL-8 and IFN-γ, in DSS-induced murine model 21, 22. Moreover, CD11b^+^CD11c^+^ DCs induced intestinal inflammation through promoting mucosal Th17 cell expansion and IL-17 responses *via* IL-23 release 14, 23, 24. Th1 differentiation were also promoted by intestinal CD11b^+^CD11c^+^ DCs 25, 26. In accordance with previous studies, our findings demonstrate that myeloid STING deletion effectively impairs the differentiation of Th1 and Th17 cells in colitis. This impairment in T helper cell differentiation can mostly be attributed to the reduced infiltration of CD11b^+^CD11c^+^DC cells associated with myeloid STING knockout. On the other hand, CD11b^-^CD11c^+^ DCs are helpful in maintaining intestinal mucosal tolerance and balancing the function of Treg cells and Th1 cells *via* producing retinoic acid, which is essential for the development of Foxp3^+^ iTreg cells, in condition of intestinal inflammation 21, 27. Although CD11b^-^CD11c^+^ DCs are usually not myeloid cell-derived DCs, our evidences showed that myeloid knockout of STING significantly inhibited CD11b^-^CD11c^+^ DC expansion with unknown mechanism. However, the proportion of Treg cells was not altered in colitic *Tmem173*^iΔmye^ mice.

Clinically, heterodimer proteins IL-12 p35/p40 and IL-23 p19/p40 have been known to link innate immunity and T helper cell-mediated adaptive immunity in excessive intestinal inflammation 28-31. Large genome-wide association studies (GWAS) also identified IL-23 receptor (IL-23R) as the susceptibility locus of UC and CD 32. Several experimental studies further demonstrated that ubiquitous transgenic expression of IL-23 p19 contributed to severe colitis 33 and knockdown of IL-12/IL23 p40 ameliorated intestinal inflammation in various experimental colitis model 34. Since patients with IBD lost response to TNF inhibitors after long-term treatment in clinical practice, the exploration of biological agents targeting IL-12 and IL-23 is emerging, such as ustekinumab, briakinumab, guselkumab, brazikumab and mirikizumab. Our results indicated that the absence of myeloid STING significantly decreased the levels of *Il12b*, *Il12a*, and *Il23a* in colitis and the deletion of STING in DMXAA-treated BMDMs/BMDCs noticeably suppressed pathways related to the IL-12 family and IL-12/IL-23 production. We further confirmed that the production of *Il12b* and *Il23a* in response to STING activation is primarily observed in BMDCs rather than BMDMs, which is in line with previous studies 35. Intriguingly, tissue-infiltrating neutrophils have also been identified as a potential source of IL-23 production 36, and the proportion of neutrophils was found potentially decreased upon myeloid STING KO in our study. Therefore, neutrophil function and neutrophil-mediated IL-23 release in the development is also worth further discovery.

Our findings further addressed more critical questions: what stimulus activate STING in BMDCs, and how does STING activation directly regulate the transcription of the IL-12 family? Emerging evidences demonstrated that the damage to mucosal barrier integrity could trigger subsequent inflammatory cascade responses, including cGAS-STING pathway sensing damage-related endogenous or bacterial dsDNA 37, 38. Contrary to the previous belief that damage-associated EV or gut microbiota-secreted c-di-GMP mediate STING activation in the colon, our research suggests that following intestinal epithelial cell damage, cell membrane rupture leads to the release of TFAM-associated mtDNA into the tissue space, ultimately mediates STING activation as DAMPS. Moreover, the association of mtDNA with certain protein scaffold and endocytosis-mediated uptake are essential for the sensing of these protein-mtDNA complex by cGAS pathway in DC cells and downstream upregulation of IL-12 family cytokines. The activation of STING can further induce the expression of type I IFNs and ISGs through the classical IRF3 pathway or mediate the phosphorylation of IKKε, leading to non-canonical crosstalk with the NF-κB pathway. However, it remains unclear whether STING activation can directly regulate the transcription of IL-12 family cytokines, and whether this regulatory mechanism depends on selective IRF3 activation or IKKε phosphorylation. Although previous studies have reported that in many inflammatory or autoimmune diseases, the activation of NF-κB is the main regulatory mechanism for the transcription of IL-12 and IL-23 39-41. However, there is a lack of experimental evidence regarding whether IRF3 can regulate the expression of IL-12 family genes. Based on our research, although it is difficult to draw conclusive and exclusive conclusions, both IRF3 and NF-κB are crucial and indispensable for the induction of IL-12 family cytokines. Our study only utilized inhibitors, which posed limitations in terms of inhibitor specificity, thus further research using genetic knockout approaches is warranted. Nonetheless, it is encouraging that we have indeed identified multiple potential binding sites for IRF3 in the promoter regions of IL-12 family genes, even more than NF-κB binding sites. This finding suggests that the activation of the downstream IRF3 pathway of STING, not only through type I IFNs, but also directly through the transcription of inflammatory cytokines, can directly participate in the regulation of adaptive immunity, opening up new avenues of research.

In summary, our study demonstrated that myeloid-specific deletion of STING in adult mice significantly ameliorated acute and chronic experimental colitis and provided protection against tumorigenesis. We also identified that damaged colonic epithelial cells released a protein-mtDNA complex, activating the STING-IRF3/NF-κB pathway in DCs. Myeloid STING knockout suppressed the expression of IL-12 family cytokines in DCs, leading to an improvement in colonic immune homeostasis by regulating Th1 and Th17 cell differentiation.

## Conclusion

Our findings comprehensively analyzed STING activation in colitis, shedding light on its origin, target cells, downstream signaling pathways, and effects on adaptive immunity. This study offers compelling evidence to support the targeted inhibition of STING activity for the treatment of colitis and related inflammatory cancer transformation, presenting new avenues for innovative therapeutic strategies.

## Supplementary Material

Supplementary materials and methods, figures.

## Abbreviations

ABX: antibiotic cocktail
AHR: aryl hydrocarbon receptor
BMDCs: bone marrow-derived dendritic cells
BMDMs: bone marrow-derived macrophages
CAC: colitis-associated carcinoma
cGAMP: 2′3′ cyclic GMP-AMP
cGAS: cyclic GMP-AMP synthase
CRC: colorectal cancer
CTL: cytotoxic lymphocyte
DAMPs: damage-associated molecular patterns
DMXAA: Vadimezan
DSS: dextran sodium sulfate
ER: endoplasmic reticulum
EtBr: Ethidium Bromide
EVs: extracellular vesicles
FBS: fetal bovine serum
GWAS: genome-wide association studies
IFN: interferon
IL-23R: IL-23 receptor
IP: immunoprecipitation
IRF3: interferon regulatory factor 3
ISGs: interferon-stimulated genes
IκBα: inhibitory subunit of nuclear factor kappa-B alpha
KO: knockout
LPS: lipopolysaccharide
MyD88: myeloid-differentiation primary response protein
NF-κB: nuclear factor kappa-B
PCA: principal component analysis
PRRs: pattern recognition receptors
STING: stimulator of interferon genes
TAM: tamoxifen
TBK1: TANK-binding kinase
TLRs: Toll-like receptors
TNF: tumor necrosis factor
5-ASA: 5-aminosalycilates
