# Supplementary material for: Intestinal epithelial damage-derived mtDNA activates STING-IL12 axis in dendritic cells to promote colitis
Source: Theranostics. 2024 Jul 16;14(11):4393–410. doi: 10.7150/thno.96184 (PMC11303083; doi:10.7150/thno.96184)
Supplement: Supplementary file 1 — Supplementary materials and methods, figures. [file thnov14p4393s1.pdf]

Supplementary materials

Materials and methods

AOM/DSS-induced colitis-associated carcinoma (CAC) model

The ablation of myeloid STING before the occurrence of inflammation was referred as *Tmem173*$^{fl/fl}$ AOM+DSS-P group and *Tmem173*$^{i\Delta mye}$ AOM+DSS-P group. 5 to 6-week-old *Tmem173*$^{fl/fl}$ mice and *Tmem173*$^{i\Delta mye}$ mice were injected intraperitoneally with 10 mg/kg Azoxymethane (AOM; Sigma, Missouri, USA) in the middle of five-time continuous tamoxifen induction. One week later, the mice were administered with 2.5% DSS for 7 days every 3 weeks. This process was repeated 3 times.

The ablation of myeloid STING after the formation of tumor was referred as *Tmem173*$^{fl/fl}$ AOM+DSS-L group and *Tmem173*$^{i\Delta mye}$ AOM+DSS-L group. Tamoxifen induction was performed after three-time DSS cycles followed by one week and the mice were sacrificed. The body weight of mice was monitored every day. Tumor tissue and colon tissue adjacent tumor were harvested at the end of the experiment.

Antibiotic cocktail experiment

C57BL/6N mice, *Tmem173*$^{fl/fl}$ mice and *Tmem173*$^{i\Delta mye}$ mice at the age of 6 to 8-week-old were orally treated with 300 μL antibiotic cocktail (ABX), supplemented with 8 g/L Ampicillin Na, 4 g/L Vancomycin HCL, 8 g/L Neomycin sulfate, and 8 g/L Metronidazole three days before DSS induction and till the end of experiment.

The assessment of disease activity index (DAI) score

DAI score was calculated as the mean value of three parameters, including weight loss, stool consistency, and gross bleeding. The weight loss was graded as follows: 0, none; 1, 1%-5%; 2, 5%-10%; 3, 10%-20%; 4, >20%. The stool consistency was graded as follows: 0, normal; 2, loose; 4, diarrhea. The gross bleeding was graded as follows: 0, absence; 2, blood tinged; 4, presence.

Histological analysis and Alcian blue-Periodic acid Schiff (AB-PAS) staining

Proximal colonic specimens were fixed in 10% neutral buffered formalin for 24 h before undergoing dehydration and embedding in paraffin. Hematoxylin and eosin

(H&E) staining was performed on 5 μm sections. The histopathological score was evaluated according to the following criteria: epithelial structure loss; crypt abscess formation; inflammatory leukocyte infiltration; goblet cell number loss; muscle layer hyperplasia. Each parameter was scored from 0 to 3. The colonic sections were also stained with AB-PAS under the guidance of corresponding staining kit (Solarbio, Beijing, China) to visualize the changes in goblet cells and mucin expression.

Quantitative real-time PCR (qPCR) analysis

Total RNA of colonic tissues was extracted and purified by lithium chloride (Sigma, Missouri, USA) to counteract the suppression of DSS on qPCR process. 1 μg of RNA was reverse transcribed using HiScript III RT SuperMix for qPCR (Vazyme, Nanjing, China). 10 ng of cDNA was then subjected to perform qPCR analysis using AceQTM Universal SYBR Qpcr Master Mix (Vazyme, Nanjing, China). Gene expressions were normalized by HPRT1. Primer sequences of qPCR will be provided as requested.

Western blot analysis

Total colonic tissues were lysed with RIPA. The concentrations of protein were measured by BCA protein assay kit (Biorigin, Beijing, China). Equal amount of protein was subjected to SDS-polyacrylamide gel and transferred to polyvinylidene fluoride (PVDF) membranes. The membranes were blocked with 5% non-fat milk powder and incubated overnight at 4 °C with various primary antibodies against p-STING (Ser366) (Cell signaling Technology, Massachusetts, USA, 50907), STING (Proteintech, Chicago, USA, 19851-1-AP), p-TBK1/NAK (Ser172) (Cell signaling Technology, Massachusetts, USA, 5483S), TBK1/NAK (Cell signaling Technology, Massachusetts, USA, 38066S), p-IRF-3 (Ser396) (Cell signaling Technology, Massachusetts, USA, 29047S), IRF-3 (Cell signaling Technology, Massachusetts, USA, 4302S), p-NF-κB P65 (ser468) (Proteintech, Chicago, USA, 82335-1-RR), NF-KB p65 (Proteintech, Chicago, USA, 10745-1-AP), IκBα (Proteintech, Chicago, USA, 10268-1-AP), IRF7 (Proteintech, Chicago, USA, 22392-1-AP). The membranes were then incubated with anti-rabbit IgG or anti-mouse IgG antibody (Proteintech, Chicago, USA). Protein levels were normalized by Beta Actin antibody (Proteintech, Chicago, USA, 66009-1-Ig) as a

control.

Immunofluorescence analysis

Colonic tissue sections were dewaxed and rehydrated through gradient alcohols. The hidden antigens were exposed by 1 mM citrate antigen retrieval solution (Beyotime, Shanghai, China) and then were blocked with 10% normal goat serum (Solarbio, Beijing, China). The sections were incubated with primary antibodies against STING (Proteintech, Chicago, USA, 19851-1-AP) and CD11B/Integrin Alpha (Proteintech, Chicago, USA, 66519-1-Ig) overnight at 4 °C. Signals were detected by goat anti-mouse IgG (H+L) Alexa Fluor Plus 488 (Thermo Fisher Scientific, Massachusetts, USA, A32723) and anti-rabbit IgG (H+L) Alexa Fluor 594 conjugate (Cell Signaling Technology, Massachusetts, USA, 8889S), and then counterstained with DAPI (Abcam, London, US). The sections were incubated with primary antibodies against STING, CD11B/Integrin Alpha and CD11/Integrin alpha (Proteintech, Chicago, USA, 60258-1-Ig) and multiple immunofluorescences were performed according to the manufacture's instruction of four-color fluorescence kit (Recordbio, Shanghai, Beijing). The co-localization of indicated targets were measured with the overlapping coefficient R calculated by plugin Colocalization Finder in software ImageJ.

RNA-sequencing and data analysis

The RNA-Sequencing was performed by Novogene Co., Ltd. (Beijing, China) as previously described [1]. Differentially expressed genes were analyzed by DESeq2 R package (version 1.20.0) with the threshold of |log2FoldChange| >= 1 and p-value <= 0.05. Gene Ontology (GO) and Kyoto Encyclopedia of Genes and Genomes (KEGG) enrichment analysis were performed using the ClusterProfiler R package (version 3.8.1) through R programming language (version 4.3.1). Gene Set Enrichment Analysis (GSEA) was determined by local version of the GSEA analysis tool http://www.broadinstitute.org/gsea/index.jsp. The immune cell infiltration analysis was performed according to RNA-seq data by local version of CIBERSORT tool and single sample gene set enrichment analysis (ssGSEA) tool. The RNA-seq results of the acute colitis experiment (Accession number GSE252100), the RNA-seq results of the BMDM

and BMDC experiments (Accession number GSE252101), and the RNA-seq results of the AOM/DSS experiment (Accession number GSE252099) are available in Gene Expression Omnibus. Each group contains three randomly selected samples for acute colitis experiment, BMDM and BMDC experiments, and AOM/DSS experiment.

Flow cytometry analysis

To analyze the innate immune responses, LP cells were stained with anti-CD45 FITC (Biolegend, California, USA, 103108), anti-CD11B Alexa Flour 700 (Biolegend, California, USA, 101222), anti-CD11C APC (Biolegend, California, USA, 117310), anti-F4/80 PE (Biolegend, California, USA, 123110) and Zombie NIR (Biolegend, California, USA, 423105). To analyze the adaptive immune responses, isolated LP cells were incubated with leukocyte activation cocktail (BD, New Jersey, USA, 550583) overnight for 10h. Then the activated LP cells were stained with anti-CD45 PerCP (Biolegend, California, USA, 103129), anti-CD3 AF488 (Biolegend, California, USA, 100210), anti-CD4 BV421 (RM4-5 clone) (Biolegend, California, USA, 100563), anti-IFN-γ AF647 (BD, New Jersey, USA, 557735), anti-IL-17 BV605 (BD, New Jersey, USA, 564169), anti-IL-4 PE (Biolegend, California, USA, 504103) and Zombie NIR (Biolegend, California, USA, 423105). The data analysis of flow cytometry was done with the FlowJo software.

The isolation and differentiation of bone marrow-derived macrophages (BMDMs) and bone marrow-derived dendritic cells (BMDCs)

Bone marrow-derived cells were isolated from the femur and tibia bones of *Tmem173*$^{fl/fl}$ mice and *Tmem173*$^{i\Delta mye}$ mice. BMDMs were differentiated from bone marrow-derived cells by culturing with fresh DPMI1640 medium containing 10% FBS, 1% penicillin-streptomycin solution (P/S), and 10% cultured supernatant of L929 cells for 7 days. Medium was replaced every two days. BMDCs were obtained from bone marrow-derived cells by culturing with fresh DPMI1640 medium containing 10% FBS, 1% P/S, 20 ng/mL GM-CSF and 10 ng/mL IL-4 for 7 days. Medium was replaced every two days. For *in vitro* studies, cells were placed into the 6-well plate and administrated with DMXAA (20, 40, 60 μg/mL), LPS (50 ng/mL), MDP (200 ng/mL), Flagellin (200

ng/mL), peptidoglycan (10 μg/mL), CM(CT), CM(TNF-α), TFAM-mtDNA complex, purified mtDNA (mtDNA isolated from fresh mouse liver using the tissue mitochondria isolation kit (Beyotime, China)), isolated DNA (TFAM-IP), and supernatant (TFAM-IP) as indicated. To be note, all doses of the other treatments in **Figure 6** are equivalent to the dose of CM (TNF-α). To be more specific, the immunoprecipitated TFAM-mtDNA complex, free mtDNA isolated from the TFAM-IP precipitate, and the supernatant of TFAM-IP were all prepared based on the same volume (500 μL) of CM (TNF-α)

The isolation and differentiation of splenetic Th1 and Th17 cells

Splenetic $CD4^+$ T cells were isolated under the guidance of EasySep™ Mouse $CD4^+CD62L^+$ T Cell Isolation Kit (Stemcell, Vancouver, Canada). Isolated $CD4^+$ T cells were incubated with anti-CD3 and anti-CD28 overnight to induce activation. To be differentiated into Th1 cells, the activated $CD4^+$ T cells were induced by 4 ng/mL IL-2, 10 ng/mL IL-12 and 10 μg/mL anti-IL-4 for three days. To be differentiated into Th17 cells, the activated $CD4^+$ T cells were induced by 50 ng/mL IL-6, 5 ng/mL TGF-β, 10 μg/mL anti-IFNγ, 10 μg/mL anti-IL-4, and 4 ng/mL IL-2 for three days. The differentiated Th1 and Th17 cells were incubated with supernatant of CT or DMXAA-treated BMDCs from *Tmem173*$^{fl/fl}$ mice or *Tmem173*$^{i\Delta mye}$ mice for 36 h. and then perform flow cytometry.

Supplementary Figure 1

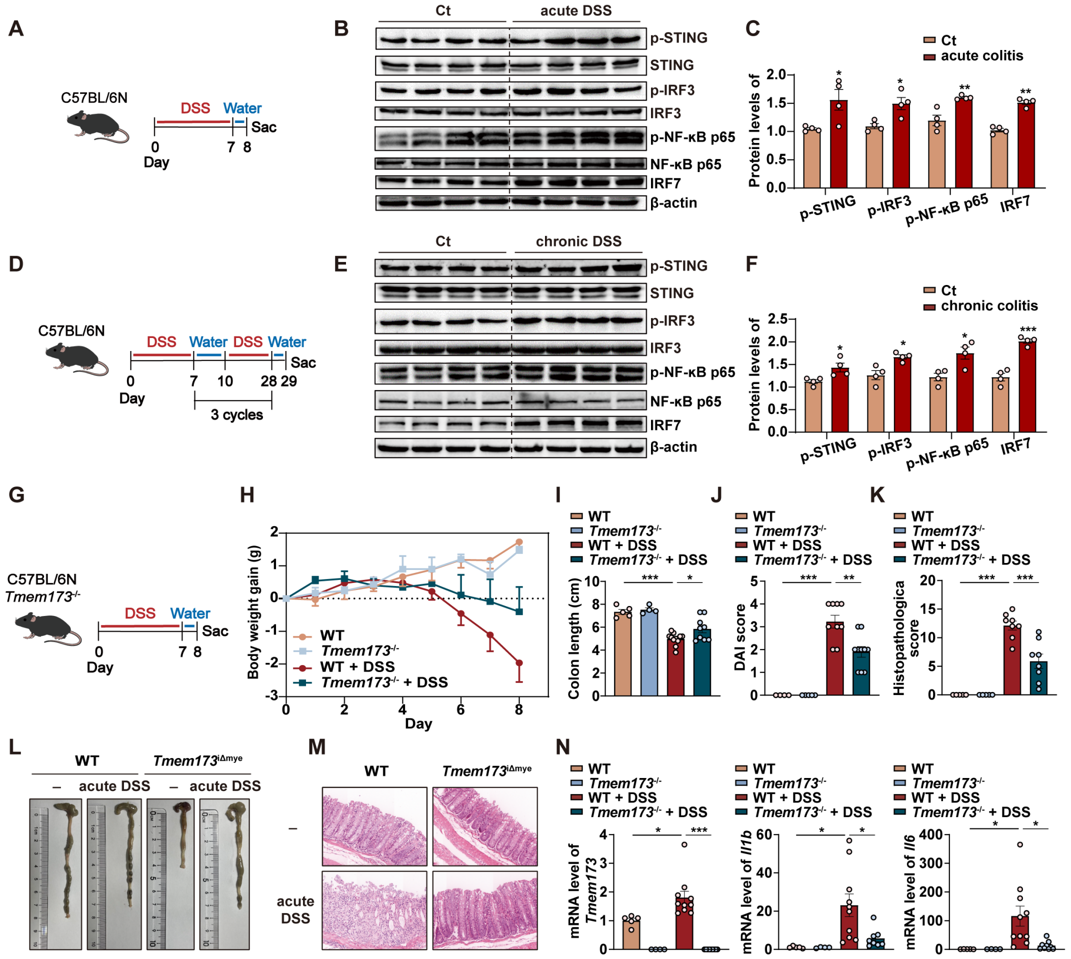

Supplementary Figure 1. (A-C) C57BL/6N mice were induced by acute DSS colitis. **(A)** Animal experimental design. **(B-C)** Representative images and quantitative analysis of immunoblotting detecting phosphorylation of STING, IRF3, and NF-κB p65 and protein level of IRF7 in colon. **(D-F)** C57BL/6N mice were induced by chronic DSS colitis. **(D)** Animal experimental design. **(E-F)** Representative images and quantitative analysis of immunoblotting detecting phosphorylation of STING, IRF3, and NF-κB p65 and protein level of IRF7 in colon. **(G-N)** C57BL/6N mice and *Tmem173*$^{-/-}$ mice were induced by acute DSS colitis. **(G)** Animal experimental design. **(H)** Body weight gain. **(I)** Colon length. **(J)** DAI score. **(K)** Histopathological score. **(L)** Representative colon pictures. **(M)** Representative H&E staining of colonic sections. **(N)** Relative mRNA levels of *Tmem173*, *Il1b*, *Il6* in colon. Scale bars, 100 μm. Values represent the mean ± S.E.M. of at least four samples in each group. Statistical significance: $^*p < 0.05$, $^{**}p < 0.01$, $^{***}p < 0.001$.

Supplementary Figure 2

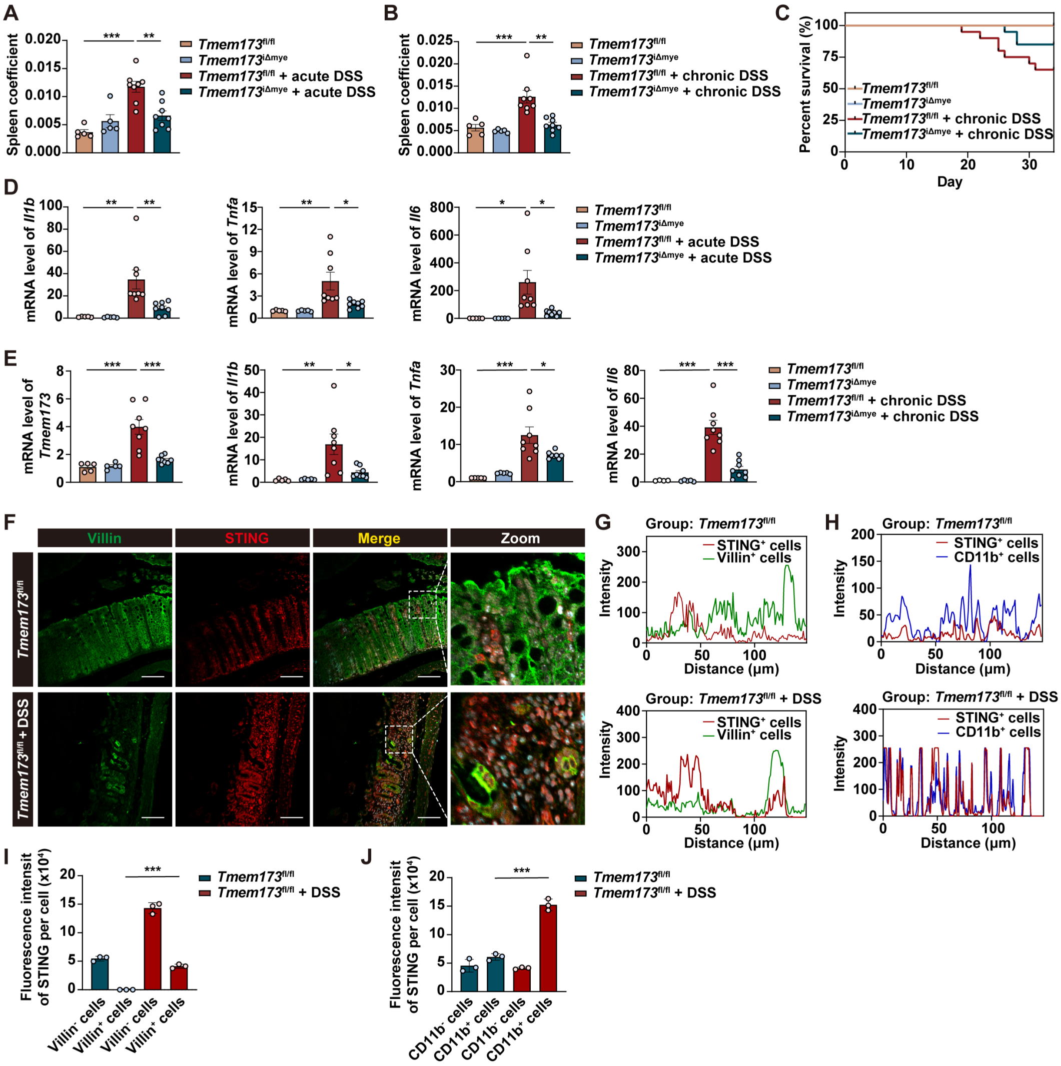

Supplementary Figure 2. (A) Spleen coefficient in acute colitis. **(B)** Spleen coefficient in chronic colitis. **(C)** Survival rate in chronic colitis. **(D)** Relative mRNA levels of *Il1b*, *Tnfa*, *Il6* in colon in acute colitis model. **(E)** Relative mRNA levels of *Tmem173*, *Il1b*, *Tnfa*, *Il6* in colon in chronic model. **(F)** Representative immunofluorescent co-staining of Villin and STING **(G)** The co-localization of Villin and STING in *Tmem173*$^{fl/fl}$ group and *Tmem173*$^{fl/fl}$ +DSS group. **(H)** The co-localization of CD11b and STING in *Tmem173*$^{fl/fl}$ group and *Tmem173*$^{fl/fl}$ +DSS group. **(I)** Mean fluorescence intensity of STING in Villin^{-} and Villin^{+} cells in *Tmem173*$^{fl/fl}$ group and *Tmem173*$^{fl/fl}$ +DSS group. **(J)** Mean fluorescence intensity of STING in CD11b^{-} and CD11b^{+} cells in *Tmem173*$^{fl/fl}$ group and *Tmem173*$^{fl/fl}$ +DSS group. Values represent the mean ± S.E.M. of at least five mice in each group. Statistical significance: $^{*}p < 0.05$, $^{**}p < 0.01$, $^{***}p < 0.001$.

Supplementary Figure 3

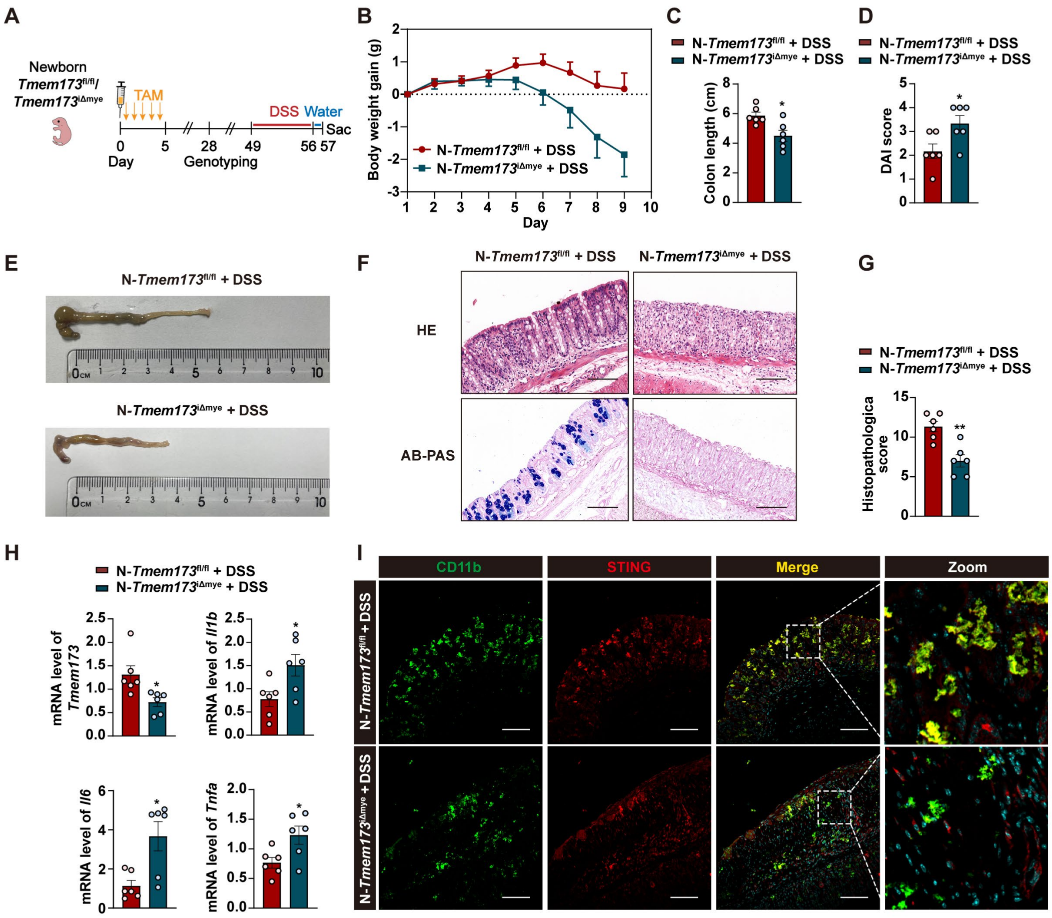

Supplementary Figure 3. Newborn *Tmem173*$^{fl/fl}$ mice and *Tmem173*$^{i\Delta mye}$ mice were induced by acute DSS colitis after TAM induction. **(A)** Animal experimental design. **(B)** Body weight gain. **(C)** Colon length. **(D)** DAI score. **(E)** Representative colon pictures. **(F)** Representative H&E and AB-PAS staining of colonic sections. **(G)** Histopathological score. **(H)** Relative mRNA levels of *Tmem173*, *Il1b*, *Il6* and *Tnfa* in colon. **(I)** Representative immunofluorescence co-staining of CD11b and STING of colonic sections. Scale bars, 100 μm. Values represent the mean ± S.E.M. of at least six mice in each group. Statistical significance: $*p < 0.05$, $**p < 0.01$.

Supplementary Figure 4

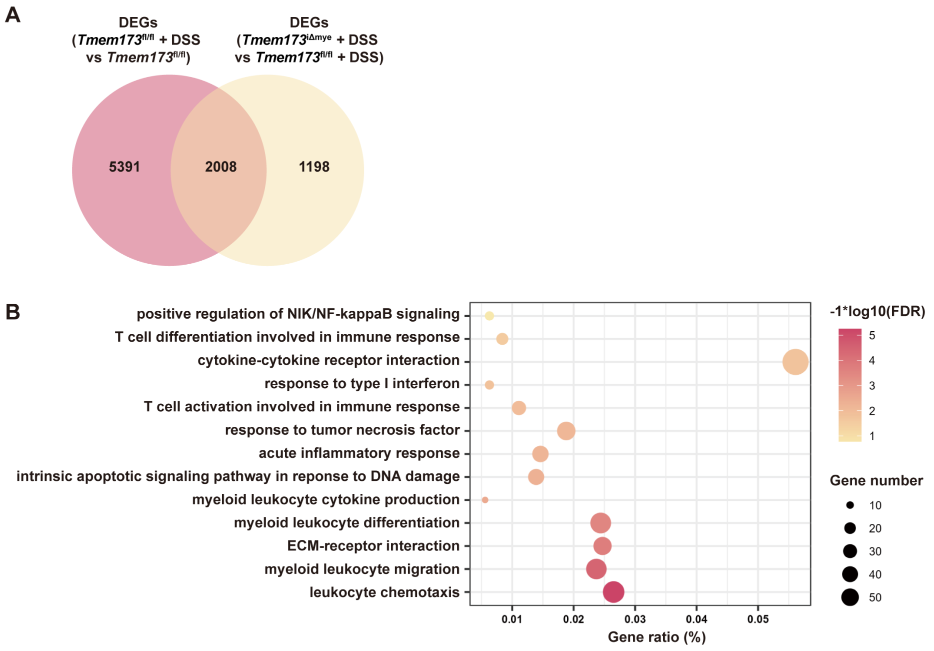

Supplementary Figure 4. (A) The overlap DEGs between *Tmem173*$^{fl/fl}$ + DSS vs. *Tmem173*$^{fl/fl}$ and *Tmem173*$^{i\Delta mye}$ + DSS vs. *Tmem173*$^{fl/fl}$ + DSS as indicated by Venn diagram. **(B)** Various inflammation-related pathways obtained by GO and KEGG enrichment analysis as indicated by bubble plot.

Supplementary Figure 5

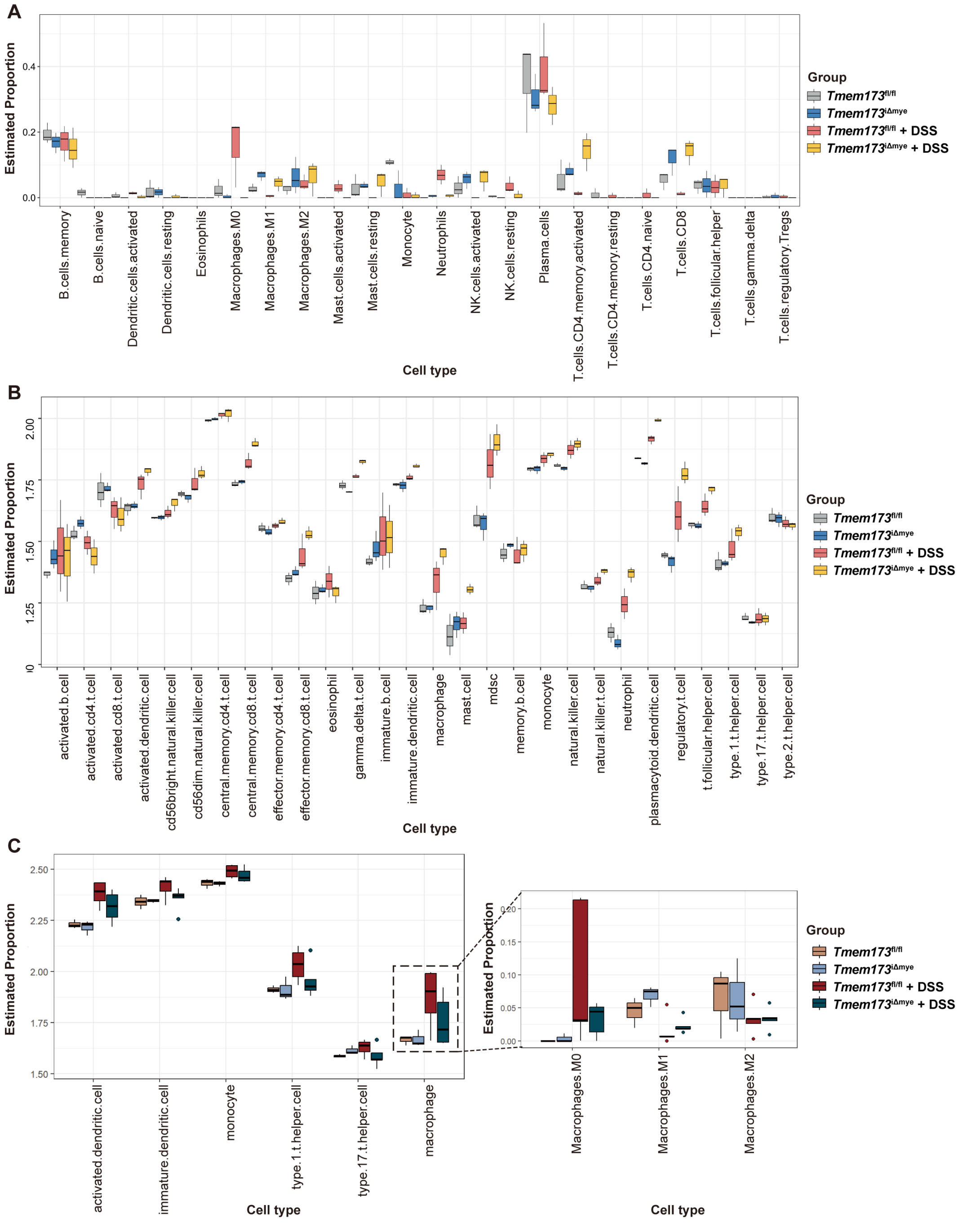

Supplementary Figure 5. (A) The immune cell infiltration analysis by CIBERSORT tool. **(B)** The immune cell infiltration analysis by ssGSEA tool. **(C)** The immune cell infiltration analysis by CIBERSORT tool and ssGSEA tool.

Supplementary Figure 6

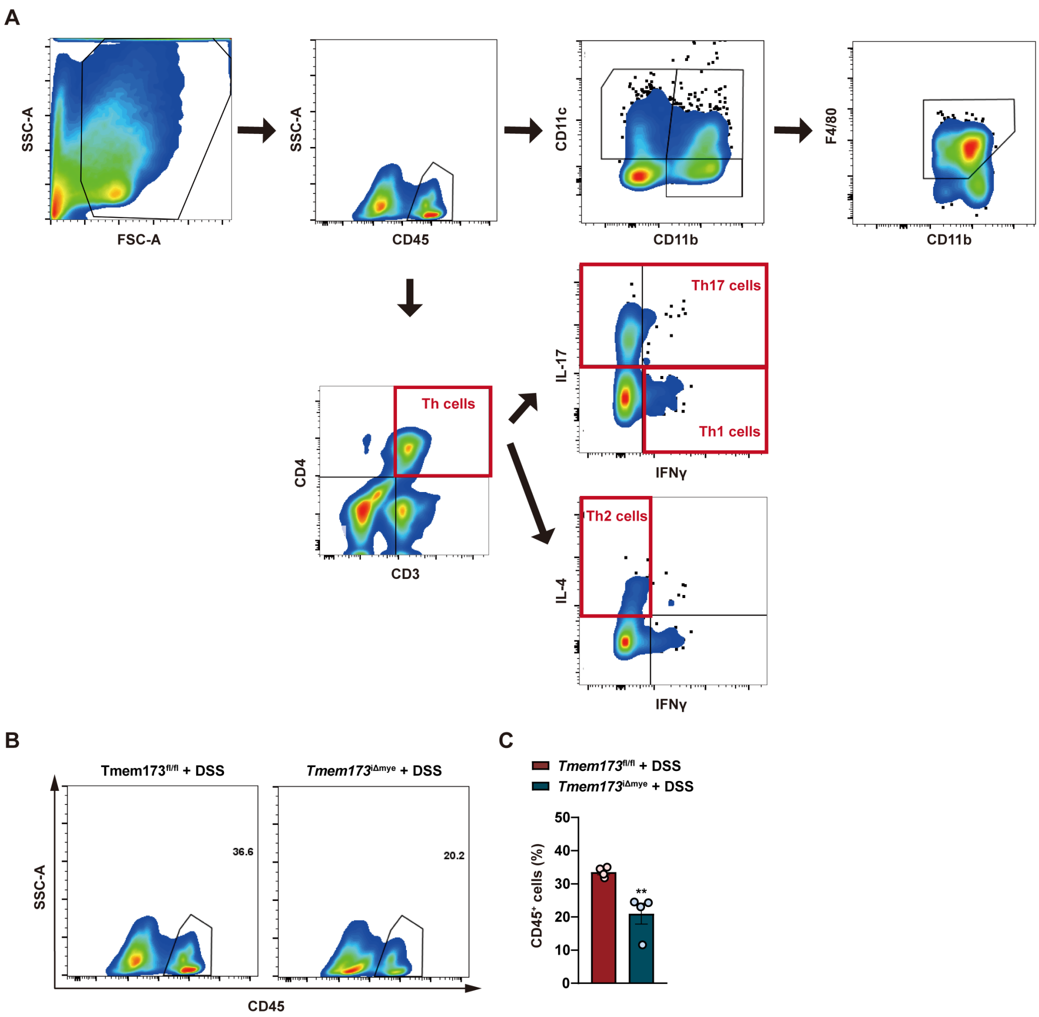

Supplementary Figure 6. (A) Sorting flow chart of flow cytometry for characterization of innate immune cells and adaptive immune cells. **(B-C)** Representative flow cytometry results and quantitative analysis of $CD45^{+}$ cells in the colonic lamina propria.

Supplementary Figure 7

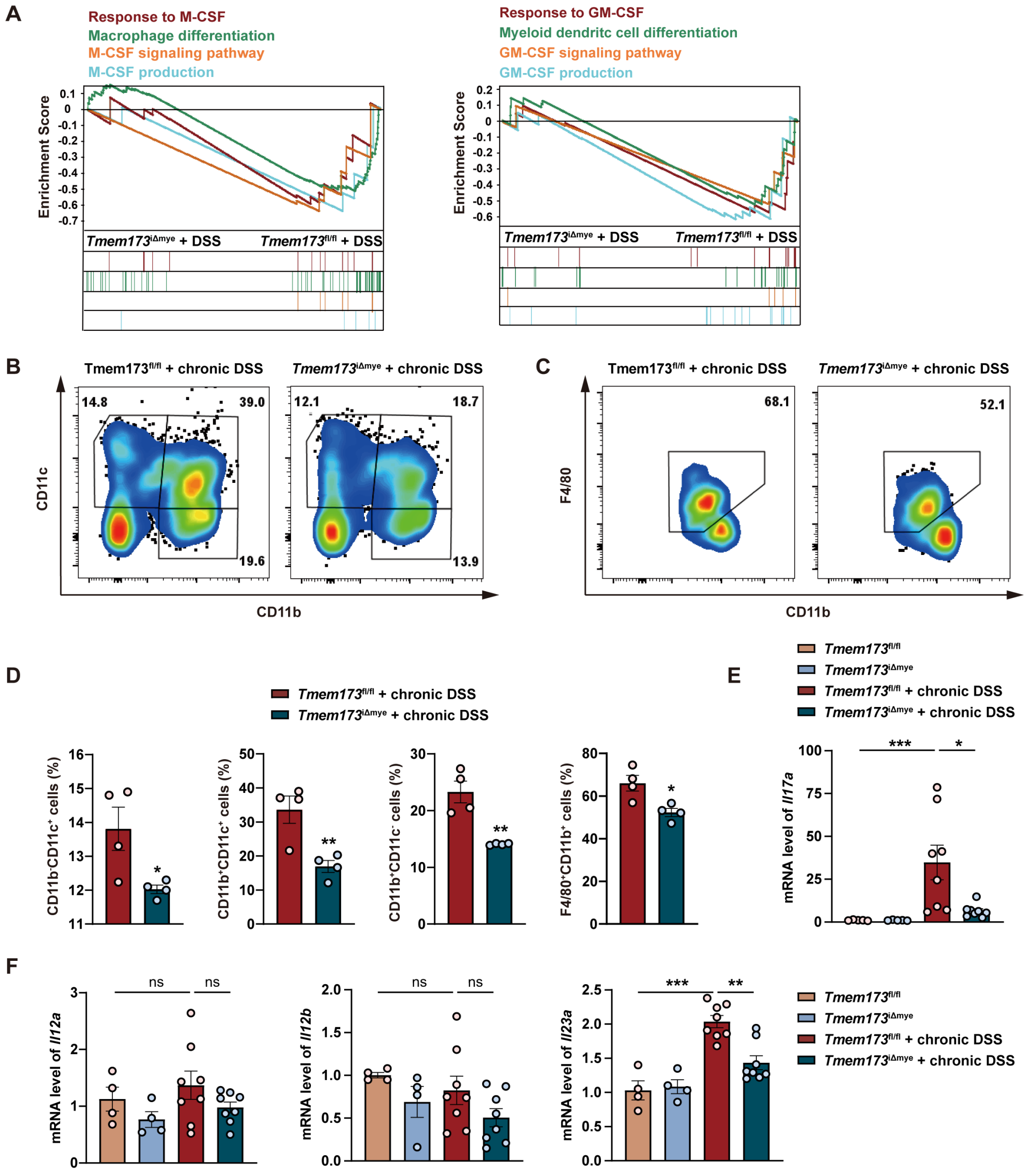

Supplementary Figure 7. (A) GSEA analysis of pathways related to macrophage and DC differentiation in *Tmem173*$^{i\Delta mye}$ + DSS *vs* *Tmem173*$^{fl/fl}$ + DSS. **(B-D)** Representative flow cytometry results and quantitative analysis of CD11b$^-$CD11c$^+$ DCs, CD11b$^+$CD11c$^+$ DCs, CD11b$^+$CD11c$^-$ monocytes and F4/80$^+$CD11b$^+$ macrophages in the colonic lamina propria in chronic DSS colitis model. **(E)** Relative mRNA levels of *Il17a* in colon in chronic DSS colitis model. **(F)** Relative mRNA levels of *Il12a*, *Il12b*, *Il23a* in colon in chronic DSS colitis model.

Supplementary Figure 8

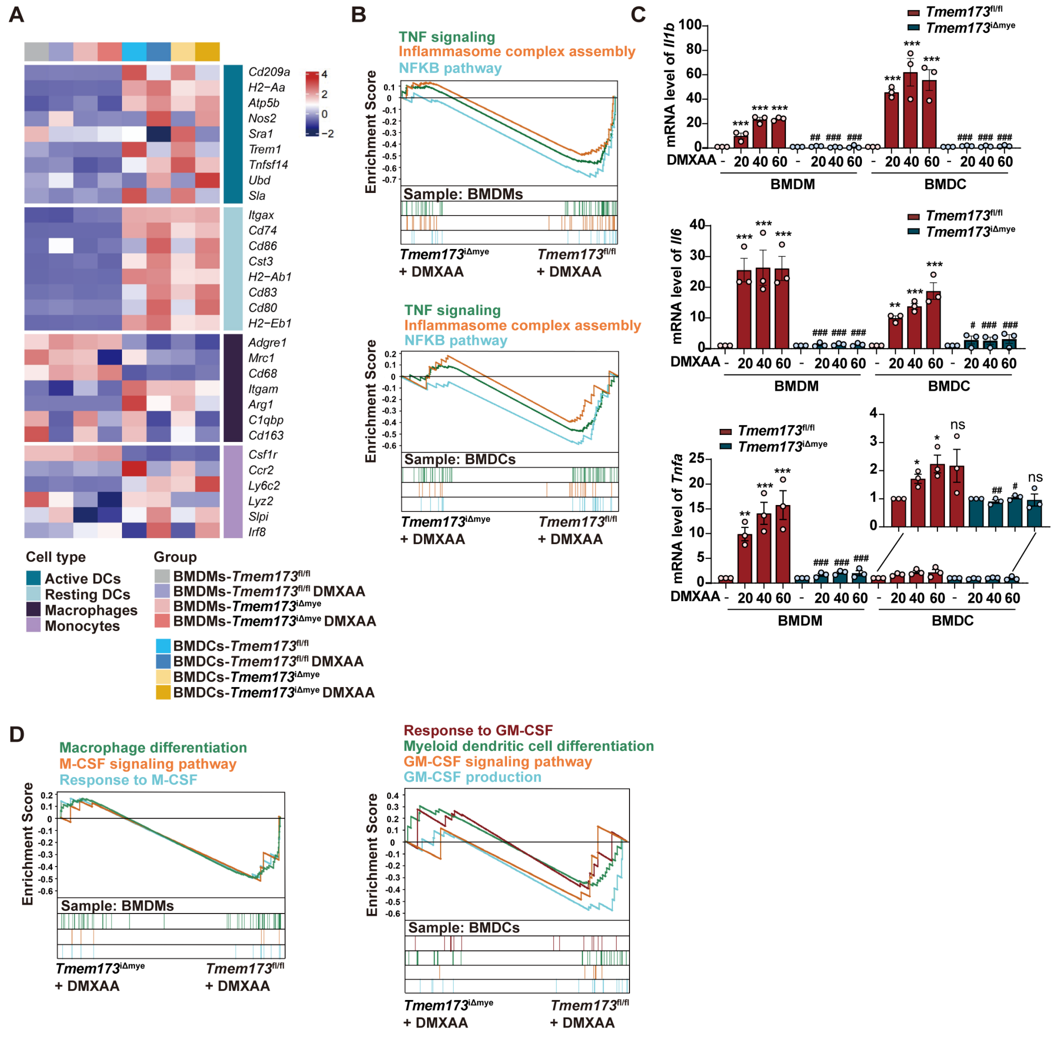

Supplementary Figure 8. (A) Gene maker expressions of active DCs, resting DCs, macrophage and monocyte in WT or STING KO BMDMs and BMDCs with or without DMXAA as indicated by heatmap. **(B)** GSEA analysis of classic inflammatory pathways in *Tmem173*$^{i\Delta mye}$ + DMXAA *vs* *Tmem173*$^{fl/fl}$ + DMXAA in BMDMs and BMDCs. **(C)** Relative mRNA levels of *Il1b*, *Il6*, and *Tnfa* in BMDMs and BMDCs. **(D)** GSEA analysis of pathways related to macrophage differentiation in *Tmem173*$^{i\Delta mye}$ + DMXAA *vs* *Tmem173*$^{fl/fl}$ + DMXAA in BMDMs, and pathways related to DC differentiation in *Tmem173*$^{i\Delta mye}$ + DMXAA *vs* *Tmem173*$^{fl/fl}$ + DMXAA in BMDCs. Values represent the mean ± S.E.M. of at least three samples in each group. Statistical significance relative to vehicle control: ns, no significant, *p < 0.05, **p < 0.01, ***p < 0.001. Statistical

significance relative to *Tmem173*$^{fl/fl}$ + DMXAA group: ns, no significant, $^{\#}p < 0.5$, $^{\#\#}p < 0.01$, $^{\#\#\#}p < 0.001$.

Supplementary Figure 9

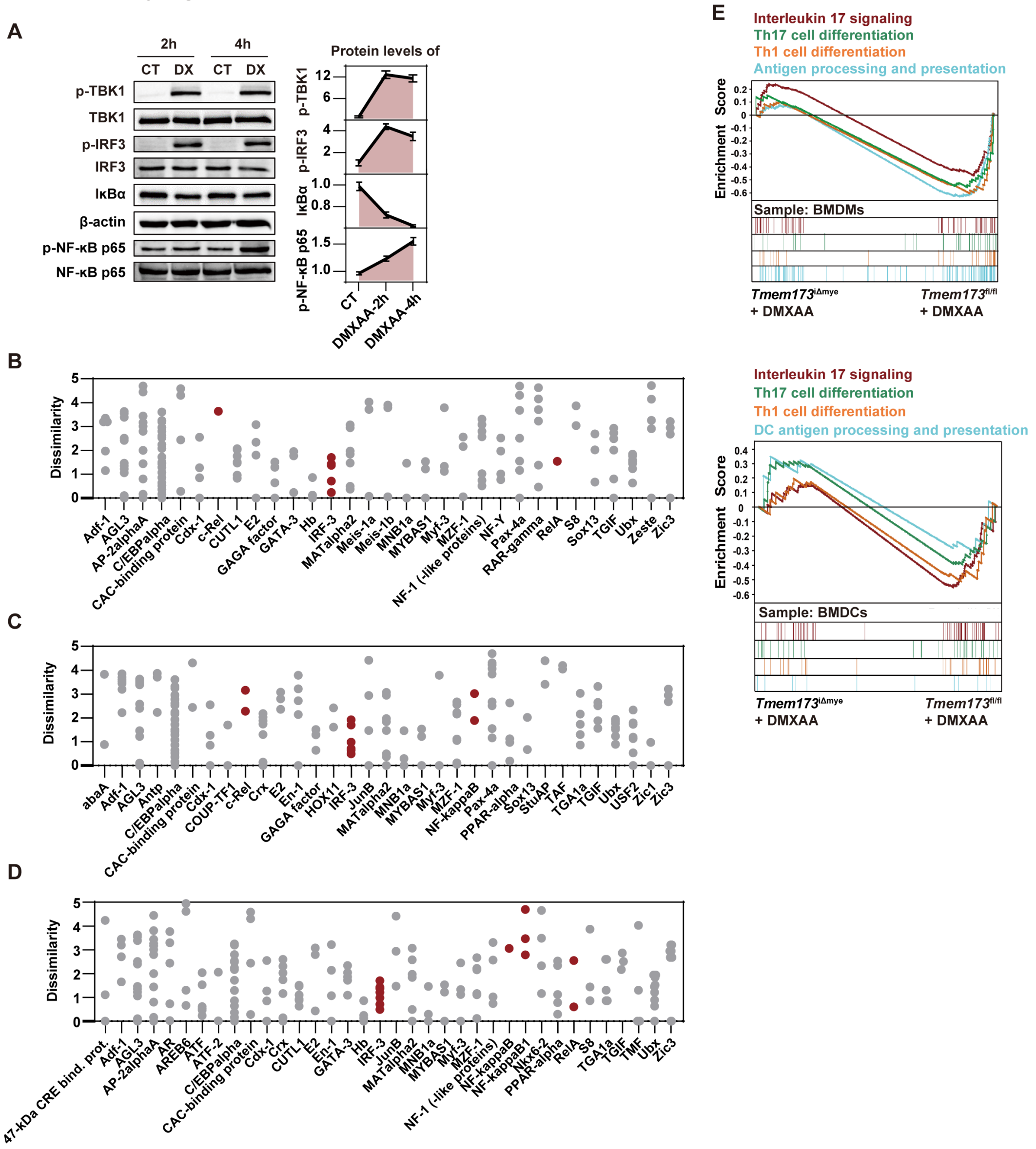

Supplementary Figure 9. (A) WT BMDCs were stimulated by vehicle control or DMXAA for 2 h and 4 h before cells were lysed for immunoblotting. Representative images and quantitative analysis of immunoblotting detecting phosphorylation of TBK1, IRF3, and NF-κB p65 and protein level of IκBα in WT BMDCs. **(B-D)** The number and dissimilarity of predicted transcription factors on promoters of IL-12 family genes (dissimilarity < 5). **(E)** GSEA analysis of pathways related to IL-17 signaling, Th1 and Th17 differentiation and antigen processing and presentation in *Tmem173*$^{i\Delta mye}$ + DMXAA *vs* *Tmem173*$^{fl/fl}$ + DMXAA in BMDMs and BMDCs.

Supplementary Figure 10

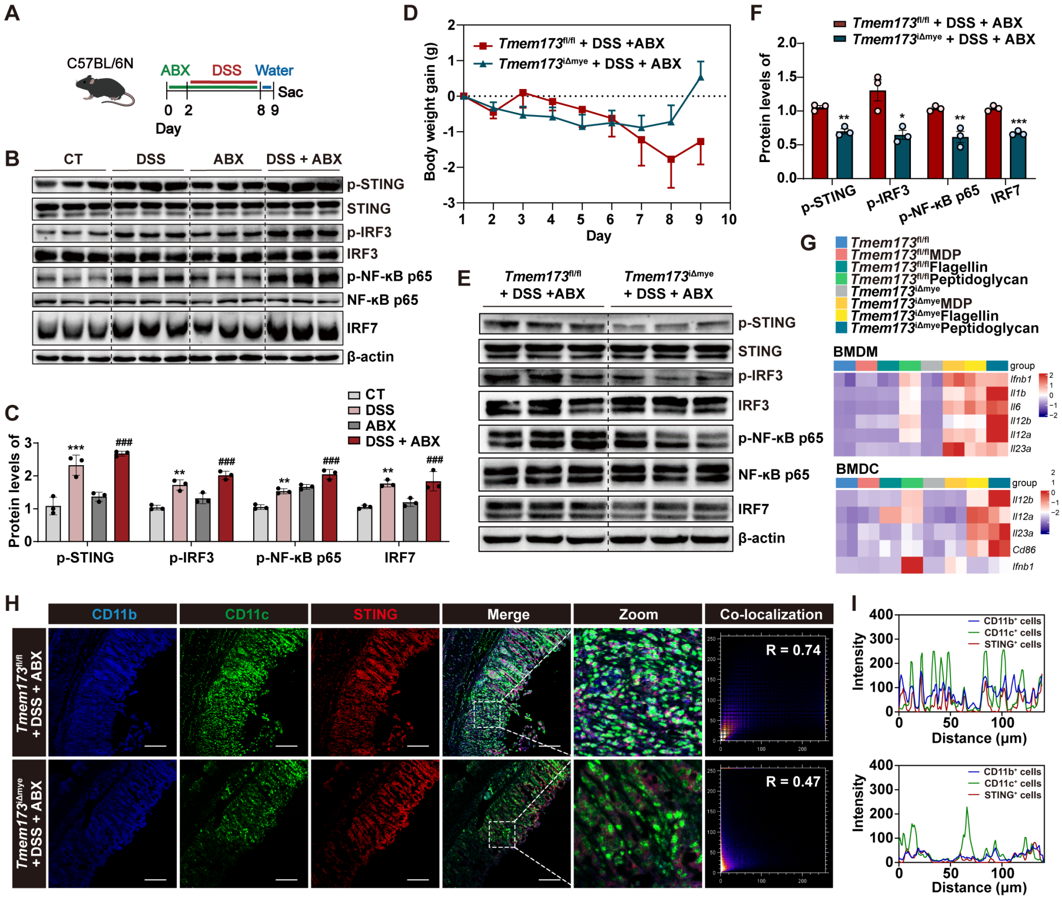

Supplementary Figure 10. (A-C) C57BL/6N mice were induced by acute DSS colitis and ABX. **(A)** Animal experimental design. **(B-C)** Representative images and quantitative analysis of immunoblotting detecting phosphorylation of STING, IRF3, and NF-κB p65 and protein level of IRF7 in colon. **(D-H)** *Tmem173*$^{fl/fl}$ mice and *Tmem173*$^{i\Delta mye}$ mice were treated by antibiotic cocktail (ABX) and then subjected to acute DSS administration. **(D)** Body weight gain. **(E-F)** Representative images and quantitative analysis of immunoblotting detecting the phosphorylation of STING, IRF3, and NF-κB p65 and the protein levels of IRF7 in the colon. **(G)** WT and STING KO BMDMs and BMDCs were treated with MDP, peptidoglycan, and flagellin for 8h. Relative mRNA levels of indicated genes are shown as a heatmap. **(H-I)** Representative immunofluorescent co-staining and quantitative analysis of CD11b, CD11c and STING of colonic sections. Scale bars, 100 μm. Values represent the mean ± S.E.M. of at least three samples in each group. Statistical significance relative to

vehicle control or *Tmem173*$^{fl/fl}$ + DSS +ABX group: *$p < 0.05$, **$p < 0.01$, ***$p < 0.001$; relative to ABX group: $^{\#}p < 0.05$, $^{\#\#}p < 0.01$, $^{\#\#\#}p < 0.001$.

Supplementary Figure 11

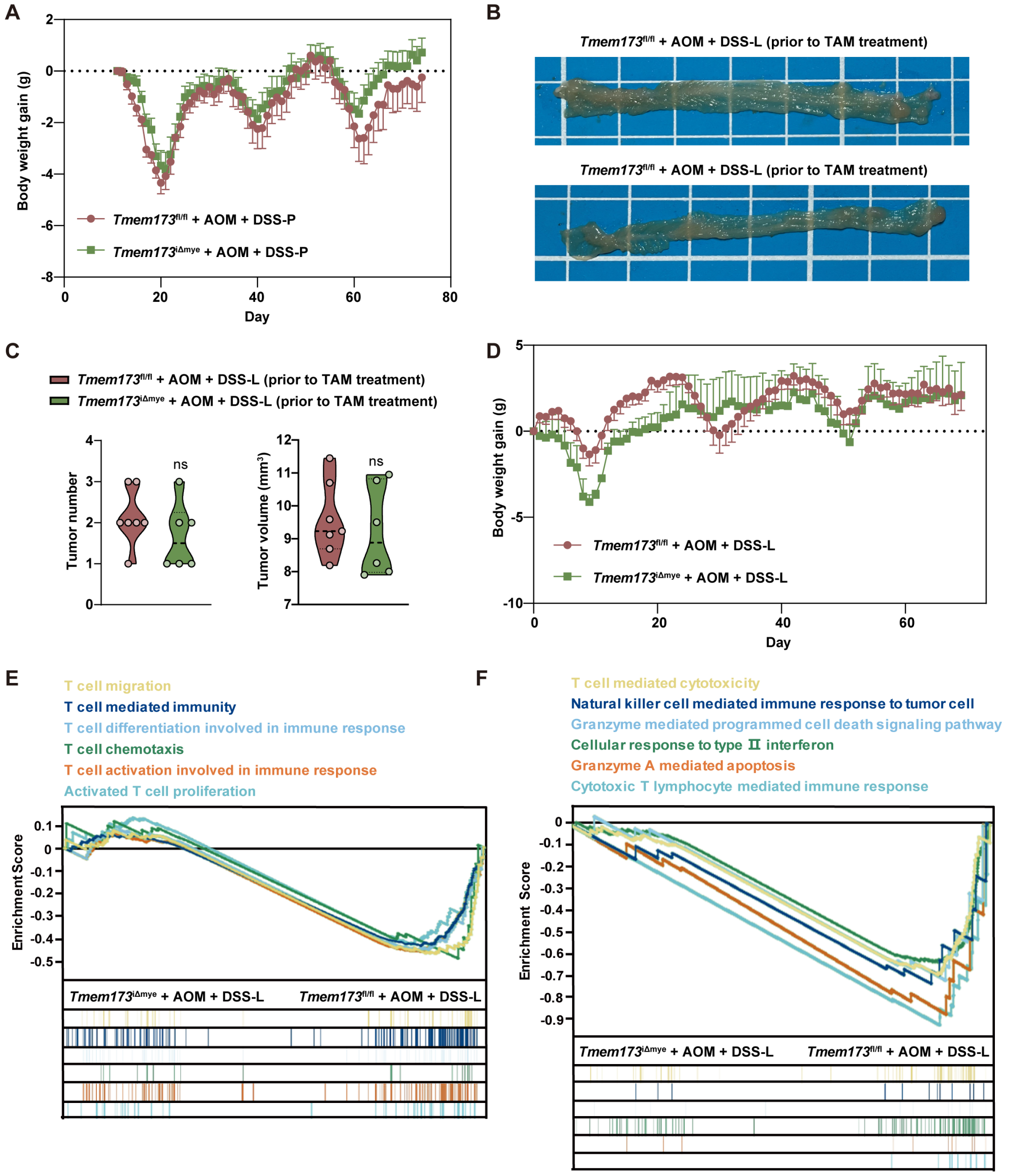

Supplementary Figure 11. (A) Body weight gain in AOM + DSS-P groups. **(B)** Representative pictures of colon samples and **(C)** the tumor number and volume in AOM + DSS-L groups before TAM induction from a preliminary experiment. **(D)** Body weight gain in AOM + DSS-L groups. **(E)** GSEA analysis of pathways related to T cell activity in *Tmem173*$^{i\Delta mye}$ + AOM + DSS-L *vs* *Tmem173*$^{fl/fl}$ + AOM + DSS-L in tumor tissues. **(F)** GSEA analysis of pathways related to tumor killing effects in *Tmem173*$^{i\Delta mye}$ + AOM + DSS-L *vs* *Tmem173*$^{fl/fl}$ + AOM + DSS-L in tumor tissues.

Supplementary Figure 12

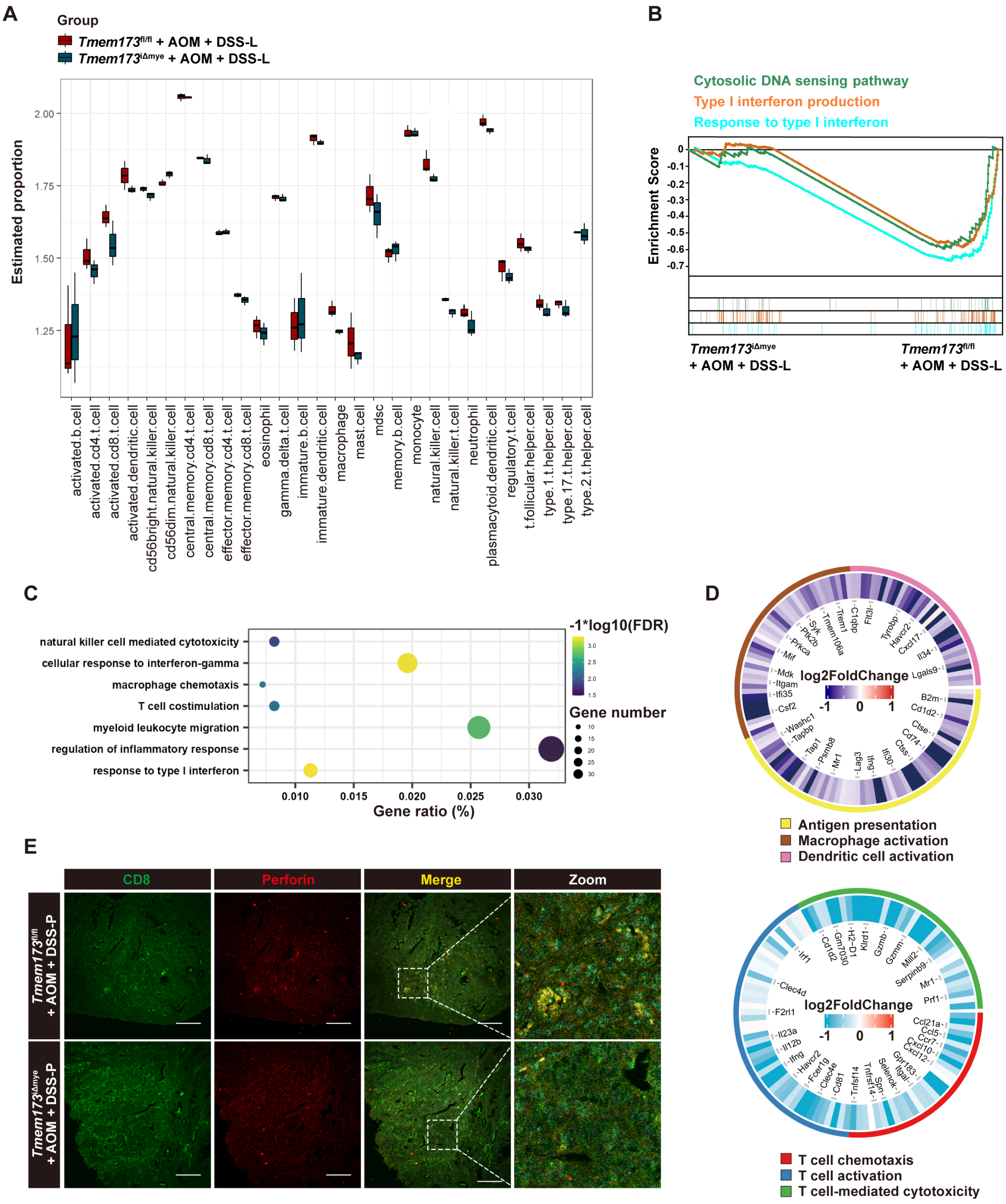

Supplementary Figure 12. (A) The immune cell infiltration analysis by ssGSEA tool based on RNA-seq data of tumor tissues. **(B)** GSEA analysis of pathways related to STING and type I IFNs in *Tmem173*$^{i\Delta mye}$ + AOM + DSS-L *vs* *Tmem173*$^{fl/fl}$ + AOM + DSS-L in tumor tissues. **(C)** Various inflammation-related pathways obtained by GO and KEGG enrichment analysis as indicated by bubble plot. **(D)** Log2FoldChange of gene expressions of innate immune and adaptive immune responses in *Tmem173*$^{i\Delta mye}$ + AOM + DSS-L *vs* *Tmem173*$^{fl/fl}$ + AOM + DSS-L in tumor tissues as indicated by

circular heatmap. **(E)** Representative immunofluorescence co-staining of CD8 and Perforin of colonic sections. Scale bars, 100 μm.
